# Confirmation of functional zones within the human subthalamic nucleus: Patterns of connectivity and sub-parcellation using diffusion weighted imaging

**DOI:** 10.1016/j.neuroimage.2011.11.082

**Published:** 2012-03

**Authors:** Christian Lambert, Ludvic Zrinzo, Zoltan Nagy, Antoine Lutti, Marwan Hariz, Thomas Foltynie, Bogdan Draganski, John Ashburner, Richard Frackowiak

**Affiliations:** a Wellcome Trust Centre for Neuroimaging, UCL Institute of Neurology, London, UK; b Unit of Functional Neurosurgery, Institute of Neurology and National Hospital for Neurology and Neurosurgery, London, UK; c LREN, Department des neurosciences cliniques - CHUV, UNIL; Lausanne, Switzerland

**Keywords:** Diffusion weighted imaging, Sub-thalamic nucleus, Segmentation, Connectivity, Hemiballismus

## Abstract

The subthalamic nucleus (STN) is a small, glutamatergic nucleus situated in the diencephalon. A critical component of normal motor function, it has become a key target for deep brain stimulation in the treatment of Parkinson's disease. Animal studies have demonstrated the existence of three functional sub-zones but these have never been shown conclusively in humans. In this work, a data driven method with diffusion weighted imaging demonstrated that three distinct clusters exist within the human STN based on brain connectivity profiles. The STN was successfully sub-parcellated into these regions, demonstrating good correspondence with that described in the animal literature. The local connectivity of each sub-region supported the hypothesis of bilateral limbic, associative and motor regions occupying the anterior, mid and posterior portions of the nucleus respectively. This study is the first to achieve in-vivo, non-invasive anatomical parcellation of the human STN into three anatomical zones within normal diagnostic scan times, which has important future implications for deep brain stimulation surgery.

## Highlights

► Three distinct sub-regions within the human STN are demonstrated in vivo using DWI. ► Limbic, associative and motor zones are labelled based on the regional connectivity. ► The findings agree with previous results from the animal literature. ► A somatotopic arrangement of STN projections to subcortical structures is shown. ► An overlap between motor STN projections and extra-STN hemiballismus is demonstrated.

## Introduction

The subthalamic nucleus (STN) is a small bi-convex structure situated in the diencephalon. Also known as the corpus Luysii, it was first described by French neurologist Jules Bernard Luys in 1865 (Luys, 1865). A critical region in the regulation of normal movement, it is also involved in limbic and associative processing (Karachi et al., 2005). The STN is a common surgical target when performing deep brain stimulation (DBS) for the symptoms of Parkinson's disease (Limousin et al., 1998). More recently it has also been proposed as a target to modulate neuropsychiatric disorders such as obsessive-compulsive disorder (OCD) (Mallet et al., 2008) and Tourette's syndrome (Martinez-Torres et al., 2009).

Located at the diencephalo-mesencephalic junction, the borders of the STN are defined by the zona incerta superiorly and postero-medially; prelemniscal radiations and postero-lateral hypothalamus anteromedially and cerebral peduncle laterally. On its inferior-most lateral surface, lies the superior aspect of the substantia nigra pars reticulata. The inferior tip lies level with the mid-point of the red nucleus (RN), the superior tip lies at the level of the posterior commissure (Naidich et al., 2009). Each nucleus is between 120 mm^3^ and 175 mm^3^ in volume (Hardman et al., 1997, 2002; Lévesque and Parent, 2005), with the majority appearing hypointense on T2*-weighted images due to the presence of iron containing neuromelanin (Dormont et al., 2004; Tribl et al., 2009; Zecca et al., 2003). It lies in a densely populated well vascularised region, and is the only excitatory glutamatergic nucleus within the basal ganglia network, projecting fibres to numerous targets (Marani et al., 2008); principally the internal pallidum, putamen, substantia nigra and thalamus. Direct cortical connections from and to the STN exist, forming the basis for the hyperdirect pathway in motor processing (Nambu et al., 1997, 2002).

Primate studies have demonstrated three functional zones within the STN: limbic, associative and sensorimotor regions residing in the anterior, mid and posterior STN respectively (Joel and Weiner, 1997; Karachi et al., 2005; Parent and Hazrati, 1995). However, these functional subdivisions of the STN have not been conclusively demonstrated in humans.

Diffusion weighted imaging (DWI) is a magnetic resonance imaging (MRI) technique that allows analysis of white matter integrity in vivo (Pierpaoli and Basser, 1996). Using probabilistic tractography, spatial distributions of white matter fibres (i.e. connectivity profiles) can be estimated for a single voxel (Behrens et al., 2003). These estimated white matter fibre pathways have been previously validated in histological studies and correspond with known anatomy (Dyrby et al., 2007). These connectivity profiles have been previously used to achieve accurate segmentation of regions not otherwise visible using conventional MRI techniques, for example the pre-motor cortices (Klein et al., 2007). The objective of this study was to explore STN connectivity and segmentation in a bottom-up, prior free fashion by proceeding stepwise through the following aims:

1. Define the normal connectivity profile within the subthalamic nucleus of healthy controls to cortical and subcortical targets.
2. Use the diffusion tractography (DT) data to estimate the number of sub-clusters within the STN.
3. Using a clustering algorithm and calculated cluster number, segment the STN into distinct regions based on the connectivity profiles.
4. Examine how cortical and sub-cortical connectivity corresponds to the calculated sub-clusters.
5. To define functional zones based on the sub-regional connectivity patterns compared to pre-existing literature.

## Methods

**Fig. 1:**
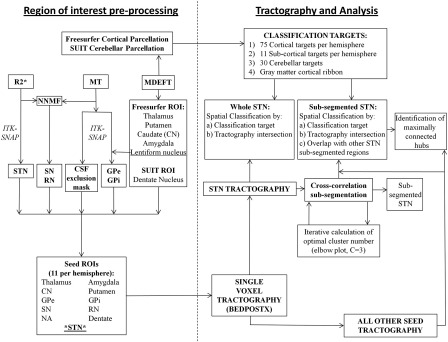
Methodological pipeline, refer to text for details. Abbreviations: CSF = Cerbrospinal fluid, CN = Caudate nucleus, GPe = External segment of the globus pallidus, GPi = Internal segment of the globus pallidus, MT = Magnetic transfer, NA = Nucleus accumbens, NNMF = Non-negative matrix factorisation, RN = Red nucleus, SN = Substantia nigra, STN = Subthalamic nucleus.

For clarity, the methodological pipeline is summarised in Fig. 1.

### Subjects

Twelve healthy right handed adults (six male, mean age males = 33.6 y, females = 34 y), underwent a single MRI scanning session at the Wellcome Trust Centre for Neuroimaging. Involvement of human volunteers was approved by the local ethics committee and each provided written informed consent prior to MRI examination.

### Image acquisition

All examinations were performed on a 3T whole-body MRI system (Trio, Siemens, Erlangen) with a 32-channel RF receive coil. The following images were acquired from each participant (see Table 1 for acquisition details): A 3D T1-weighted modified driven equilibrium Fourier transform (MDEFT) image (Deichmann, 2006), two identical DWI datasets and several images to estimate parametric T1 and T2* maps (“multispectral sequence”) (Lutti et al., 2010). The multispectral acquisition protocol produces magnetic transfer (MT), T1 and Proton Density (PD) weighted images (1 mm isotropic resolution, total acquisition time 19 min) (Helms et al., 2008). For each subject quantitative MT, T1 and R2* (1/T2*) maps were extracted from the images using in-house MATLAB code. B1 RF field maps (4 mm isotropic resolution) were acquired using a 3D EPI SE/STE method (Lutti et al., 2010) and used to correct the T1 maps for RF transmit field inhomogeneity effects.

### Diffusion preprocessing

The two diffusion acquisitions were eddy current corrected and averaged using FSL (FIMRIB, Oxford, England). Skull stripping was performed by applying brain masks derived from the images with b = 100 s/mm^2^ using the unified segmentation within SPM8 (http://www.fil.ion.ucl.ac.uk/spm/) (Ashburner and Friston, 2005). The skull stripped brain was visually checked for errors prior to any further processing. Initial tensor estimation was performed using DTIfit in FSL (http://www.fmrib.ox.ac.uk/fsl/), and the results visualised prior to tensor estimation using a ball-and-stick model in BEDPOSTX (Behrens et al., 2007). Registration to structural space was also performed using FLIRT in FSL. For each subject, the resulting structural to diffusion registration was manually checked to ensure satisfactory alignment, with particular attention paid to the regional borders of the STN.

### Brain parcellation

The major cortical and subcortical structures were initially parcellated using the Freesurfer recon-all pipeline (Fischl et al., 2004)(http://surfer.nmr.mgh.harvard.edu/). This produced 75 cortical regions per hemisphere that were divided according to the Destrieux 2009 atlas (Destrieux et al., 2010). Using this pipeline, six subcortical regions per hemisphere were also defined (nucleus accumbens, caudate, putamen, pallidum, thalamus, and amygdala). The pallidal regions were then subdivided using ITK-SNAP (http://www.itksnap.org) (Yushkevich et al., 2006) “draw over label” function, into the internal (GPi) and external globus pallidus (GPe) using the medial medullary lamina as the boundary between the two. This was clearly demarcated on the MT images. The substantia nigra and red nucleus were defined by generating population tissue probability maps for six tissue classes using a novel segmentation algorithm developed in-house. For this, the group MT and R2* images were normalised using DARTEL in SPM (Ashburner, 2007) to MNI space at 1 mm^3^ resolution and the corresponding Jacobian determinants calculated. A modified version of a mixture of Gaussians model was then fitted to the collection of spatially normalised images, accounting for regional expansion or contraction by incorporating the Jacobian determinants into the computations. Because the images were quantitative, the intensity distributions of each tissue class were assumed to be identical for all subjects and modelled as a bivariate Gaussian. In addition, the model assumed that at any location in all of the spatially normalised images, there were the same prior probabilities in observing the various tissue types. These prior probabilities were estimated from the model and served as population tissue probability maps. For each region of interest (ROI), the regional probability maps covering our areas of interest were extracted using previously defined spatial priors from n = 10 subjects which were dilated using 5 mm Gaussian smoothing and binarised at a threshold of 0.01. For each subject, the labels were assigned based on the maximum joint probability between the population spatial tissue probability maps, and individually calculated voxel intensity probability density functions (for white matter and ROI).

The cerebellum was parcellated into 28 regions and two dentate nuclei using the SUIT toolbox (http://www.icn.ucl.ac.uk/motorcontrol/imaging/suit.htm) (Diedrichsen, 2006; Diedrichsen et al., 2009) in SPM8. A CSF exclusion mask that included the ventricles was generated for each subject using ITK-SNAP snake function within the CSF spaces on the MT images. Finally all the labelled ROIs were moved back to subject space using the SPM-Deformations tool.

The pedunculopontine nuclei were not analysed as target or seed regions for tractography in this current study. This was because they could not be clearly delineated from the closely associated lemniscal system or superior cerebellar peduncle on any of the acquired images.

### STN identification

Due to the variability in STN position and orientation, direct visualisation is the most accurate method to identify the structure (Ashkan et al., 2007; Hariz et al., 2003; Stancanello et al., 2008). Using R2* images, the hyperintense region of the STN was manually segmented using ITK-SNAP software (Supplementary Material 1) by the investigator (CL). Surrounding anatomical structures were visualised simultaneously on the MT images using ITK-SNAP multisession function, and were used to aid identification of the superior and lateral boundaries of the STN (as described in Supplementary Material 1 legend). To ascertain reproducibility, a functional neurosurgeon (LZ) defined STN pairs on four of the subjects, providing eight STN volumes to ascertain reliability of STN delineation.

### Probabilistic tractography

Probabilistic tractography was performed using FSL probtrackX software (Behrens et al., 2007). Within each of the ROIs, unconstrained whole brain tractography was performed for every individual voxel. Each voxel was sampled 5000 times with a curvature threshold of 0.2, modelling two fibres per voxel and applying a CSF exclusion mask applied. Tractography was run from 11 seed regions per hemisphere (22 in total). These were the putamen, nucleus accumbens (NA), caudate (CN), GPi, GPe, thalamus, STN, red nucleus (RN), substantia nigra (SN), amygdala and dentate nucleus. Using MATLAB 2009a, the raw, single voxel tractography distributions were initially analysed by calculating their maximum connectivity values with the 202 parcellated regions including bilateral cortical (N = 150), sub-cortical (N = 22) and cerebellar (N = 30) targets, and then defining the frequency with which these connections existed in each sub-region across the group.

### Analysis

All analysis was performed using MATLAB 2009a.

### Literature review

**Fig. 2:**
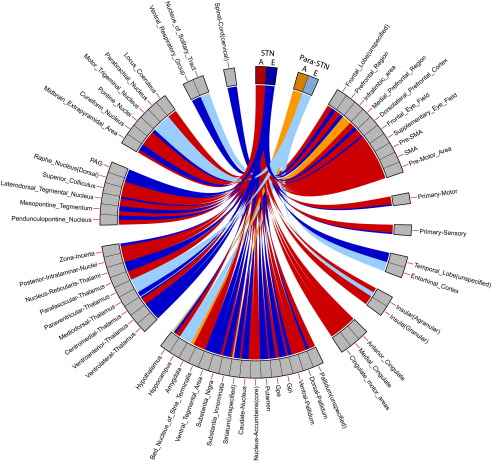
Circular ideogram summarising a literature review of the STN afferent and efferent connectivity in mammals. For comparison, reported parasubthalamic connections are included but uniquely labelled. STN afferents are shown in red and efferents in blue. Para-STN afferents are in orange and efferents in pale blue. The width of each connecting ribbon represents the normalised proportion (as a percentage) of the respective connections. Refer to the Methods for details on how this was calculated. A summary of the data and articles reviewed can be found in Supplementary Material 2.

A PUBMED search for the term “subthalamic nucleus” was performed and identified 3771 publications. Any report using or reviewing tract tracing was obtained and reviewed, and any connections with the STN documented. The inclusion criteria were reports written in English (n = 4 excluded (Makoev, 1981; L'vovich, 1978; Oleshko, 1985; Sotnichenko and Istomina, 1984)), only involving mammals (n = 3 excluded (Belekhova, 1991; Brauth et al., 1978; Martínez-Marcos et al., 1999)). Four articles were unobtainable (Jackson and Crossman, 1981a; Nauta and Cole, 1974; Nauta and Domesick, 1984; Saper and Loewy, 1982). Probabilistic tractography studies were not included (Aravamuthan et al., 2007) in this review to attempt to generate a gold standard baseline of expected connectivity. Only direct pathways were considered, as these are the ones that are detectable using DTI. A recently reported disynaptic projection to the cerebellum (Bostan et al., 2010) was classified according to the intermediate direct projection to the pontine nuclei. Also, several papers also define a parasubthalamic nucleus (Goto and Swanson, 2004; Mascaro et al., 2009), that lies medial border of the subthalamic nucleus at the posterior level of the lateral hypothalamus (Paxinos and Watson, 2007). This structure has been described in rodents but not primates. However, to better understand the tractography results, these have been included in the review and specifically labelled as separate categories. A total of 130 articles from 1947 to 2011 were reviewed and the information has been summarised in an ideogram shown in Fig. 2 using the circular graph making software Circos (http://circos.ca/) (Krzywinski et al., 2009). Each connection has been coloured according to afferent (red) or efferent (blue) connectivity. Parasubthalamic nucleus connections are differentiated using orange or light-blue. The width of each ribbon indicates the normalised proportion of reports as a percentage. Normalised proportion (*P*_*n*_) was calculated first calculating the number of reports (*N*) detailing a regional connection (*c*) as a percentage of the total number of reports (*T*) for that particular type of connection (*i*), where *i* is either the afferents or efferents, where the parasubthalamic afferents and efferents were added to the STN. Four connection classes (*k*) were considered, namely STN afferents and efferents, and parasubthalamic afferents and efferents. This was primarily done to try and account for the fact that there are far more STN afferents reported (n = 226) than efferents (n = 184):

$$P_{k}=100*\left(\frac{N_{\mathrm{ck}}}{\sum_{c=1}^{c=T}N_{\mathrm{ci}}}\right)$$

$$P_{n}=100*\left(\frac{P_{\mathrm{ck}}}{\sum_{k=1}^{k=4}P_{\mathrm{ck}}}\right)\text{.}$$

*Pn* is intended to reflect the pattern of afferents and efferents between the STN and a region of interest. Supplementary Material 2 includes a table giving the proportions (*P*) for each region and lists all papers used in the review.

### Seed connectivity

Using a probability index of connectivity (PICo) threshold of 1%, the connectivity between each STN to the ipsilateral sub-cortical ROIs (all seed images plus hippocampi) was defined and images for each sub-cortical ROI demonstrating the spatial location of connecting voxels were generated. For the seed ROIs, this was additionally refined by defining the voxels that connected from the ROI to the ipsilateral STN, and the product of the two images used for further analysis. These images were warped into 1 mm MNI space using DARTEL in SPM8 (Ashburner, 2007) and the data were averaged to create connection probability maps for each structure. The cortical connectivity was analysed by applying a previously described method of STN-cortical thresholding (Aron et al., 2007). Specifically, the maximal connectivity value from the STN to the cortex was defined for each subject, and then a threshold of 2% maximum cortical value was applied to the individual tractography distributions. On average, this corresponded to a PICo value of 19 in arbitrary units. This method was chosen for two reasons. First, it provided a means of distance correcting the PICo values, which are known to decrease with distance from the target. Second, as in Aron et al., the intention was to examine the spatial distribution of STN-cortical intersections, relying on exact overlap of fibres across subjects in standard space, which tends be overly conservative. These images were binarised, transformed to common space, and then group averaged. For every region, the percentage of subjects with connectivity within a region was defined. Connections present in > 25% were reported. Additionally, the corresponding tractography-cortical intersections were visualised by thresholding the group tractography distribution at 25% and multiplying it with a grey-matter mask. The results were rendered in ITK-SNAP and resultant mesh smoothed for illustrative purposes with an 8 mm FWHM Gaussian kernel.

### STN sub-segmentation

The raw, full brain PICo distributions for each STN voxel were binarised at a threshold of 1%. Using the binarised distributions at 1 mm isotropic resolution, a cross correlation matrix was generated according to previously described methods, but with no down-sampling or binning of data (Johansen-Berg et al., 2005). The correlation matrices were clustered using a cluster-Ward-linkage algorithm. Ward's linkage is an agglomerative, hierarchical clustering method that attempts to minimise the sum of squares error at each stage, and can be implemented via the *linkage* function in MATLAB.

Previous DT studies have specified the number of clusters based on a prior hypothesis formed from the literature. In the STN, animal studies suggest that three regions may be present, however, there is little in the way of human data to support this. Therefore a data driven method to define the number of clusters was tested. To define the optimal number of clusters, the algorithm was run from two to twenty clusters for each subject. At each point the percentage ratio between the new within cluster sum of squares and total sum of squares was calculated. This was performed as follows. Taking the two dimensional connectivity correlation matrix(*C*), consisting of *N* voxels, the total sum of square errors (*T*_*SSE*_) was calculated:

$$T_{\mathrm{SSE}}=\sum_{i=1}^{N}\sum_{j=1}^{N}{\left(C_{\mathrm{ij}}-\frac{1}{N}\sum_{m=1}^{N}C_{\mathrm{mj}}\right)}^{2}\text{.}$$

Given that *K* is the total number of clusters at each point, for each subcluster *k*, *P* = 1 if a voxel belongs to that cluster and 0 otherwise, where *k* ≤ *K* and *K* ≥ 2, the within clusters sum of square errors (*W*_*SSE*_) is:

$$W_{\mathrm{SSE}}=\sum_{k=1}^{K}\sum_{i=1}^{N}P_{\mathrm{ik}}\sum_{j=1}^{N}{\left(C_{\mathrm{ij}}-\frac{\sum_{m}^{N}P_{\mathrm{mk}}C_{\mathrm{mj}}}{\sum_{m}^{N}P_{\mathrm{mk}}}\right)}^{2}\text{.}$$

The fractional variance explained for each selected total cluster number is given by:

$$1-\frac{W_{\mathrm{SSE}}}{T_{\mathrm{SSE}}}\text{.}$$

The fractional variance explained was plotted for each individual, and the elbow criterion (Timm, 2002) used to support the optimal cluster number. Further analysis was performed using the calculated optimal cluster number that occurred most frequently. The “elbow” was assessed visually (Young et al., 2006), and defined as the point in the plot where a sharp, angled change occurred after which the fractional variance explained changed very little with the addition of more clusters, indicating that minimal additional information is gained after this point. An example of this is shown in Fig. 4. This approach avoided bias both in the selection of cluster number and also the delineation of cluster boundaries, as each was generated automatically through this method.

For each sub-parcellated STN region, the connectivity values to and from each clustered sub-region were re-calculated for every individual and all the results averaged in standard space. The process was repeated for each STN sub-region across all ROI tractography distributions. If connectivity was found in > 50% of subjects, the spatial distribution of cluster sub-cortical connectivity was visualised by rendering the regions that overlapped in at least 25% of subjects, defining each according to which cluster had the maximum group connectivity probability value. 25% was chosen for visualisation due to the loss in spatial resolution and tendency for non-contiguous clusters to result from the smoothing of rendered meshes. Corresponding colour-coded renderings were created using MATLAB and ITK-SNAP with 8 mm mesh smoothing. Overlap regions were also analysed. This was done by initially warping each sub-regions tractography profiles to standard space and creating six average images. These were the anterior, mid and posterior cluster tracts for the left and right sides. These were used for classifying each brain voxel within a ROI according to the combination of these summary distributions that connected with it (using the 1% threshold for all subcortical structures, and 2% maximum connectivity for the cortical and cerebellar hemisphere results).

### Visualisation of results

**Fig. 3:**
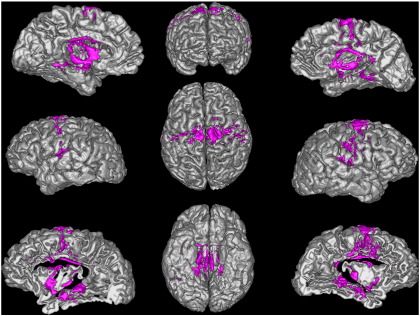
Cortical regions of common ipsilateral STN connectivity across the group. Dark grey regions demonstrate cortico-tractography intersections. Each hemisphere is shown on the corresponding side. Views, clockwise from top-left: Left medial surface, anterior surface, right medial surface, right lateral surface, right lateral insula, inferior surface, left lateral insula, left lateral surface. Superior surface central image. See Table 1 for details of cortical regions.

Three-dimensional renderings have been generated using ITK-SNAP to aid the interpretation of the results (Figs. 3, 5 and Supplementary Material 3). These have all been smoothed for illustrative purposes with an 0.8 mm kernel. This can cause the clusters to appear smaller, and non-contiguous in the rendered images compared to the original data. Additionally the cortical renderings (Fig. 2) rely on full intersections between the tractography and grey matter mask, hence all the regions reported in Table 2 may not be visible on the rendered images. For this reason we refer to the connectivity visualised in the cortical renderings as “cortico-tractography intersections”, and the results should be interpreted in conjunction with Table 2.

## Results

### STN Volume

The average STN volume was 155.4 mm^3^ (right, standard deviation = 24.9 mm^3^) and 155 mm^3^ (left, standard deviation = 28.6 mm^3^). There was no significant difference between STN volumes on the left and right side (p > 0.05). Analysis of reliability between observers (Cronbach's alpha) for the manual delineation of the STN was 0.96. Reported STN volumes are variable, and the values here fall within the range cited by Lévesque and Parent (2005) (175 mm^3^ ± 20.3). A likely explanation for the differences is the variation in iron distribution, which is known to decrease in the posterior portion of the nucleus, leading to reduced MR contrast.

### Literature review

The literature review of STN tract tracing is summarised in Fig. 2 and in the Supplementary Material 2. It provides a basis with which to interpret the results listed below.

### Global cortical connectivity pattern

A summary of cortical and subcortical connectivity with the STN is shown in Table 2 and Fig. 3. In discussing these results, a distinction is drawn between weak and strong connectivity. Weak connectivity is defined as being present in 25–50% of subjects whereas strong connectivity is present in more than 50% of subjects.

#### Subcortical

All the sub-cortical regions analysed demonstrated strong connectivity to the entire STN. Interestingly two distinct clusters in each hippocampus were robustly identified in the majority (> 75%) of subjects.

#### Cortical

Bilateral cortical regions that demonstrated strong connectivity to the entire ipsilateral STN across subjects were the precentral, medial and superior frontal gyri. These are the regions in which the motor and pre-motor areas reside, and include the origin of the hyperdirect pathway fibres (Nambu et al., 1997). The tractography-cortical intersections show clustered connections to these regions, which agrees with current models of STN function. Additionally, two distinct clusters within each insula were reliably identified across subjects, one anterior and one posterior. A section through the cortex revealing each insula is shown in Fig. 3, demonstrating the two clusters. Table 2 provides a breakdown of bilateral STN ipsilateral cortical connectivity. Though largely symmetric, asymmetrical projections (defined as only being present in one hemisphere) were identified in two regions with weak connections (33% of subjects); the temporal pole on the left and the orbital gyrus on the right.

#### Cerebellum

There were no significant connections to any part of the cerebellum that survived thresholding. The maximum connecting value in arbitrary PICo units was 9.

### Sub-parcellation of the STN

**Fig. 4:**
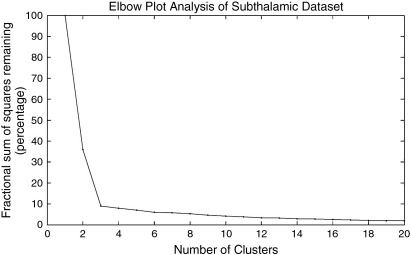
Example elbow plot demonstrating optimal cluster number given probabilistic tractography data for a single STN. Clear “elbow” shown at n = 3 clusters.

**Fig. 5:**
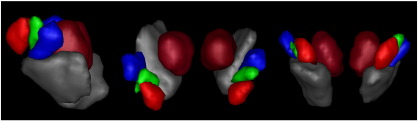
Rendering of group averaged sub-segmented STN regions. The voxel borders of the group were defined according to which cluster they were maximally assigned to across all subjects. Anterior “limbic” cluster = red, middle “associative” cluster = green, posterior “motor” cluster = blue. Left lateral, superior and anterior views demonstrated above. The relationship to group averaged renderings of the red nuclei (dark red) and substantia nigra (grey) are shown. Similar segmentation patterns were achieved on an individual subject basis.

For each STN a whole brain binary connectivity matrix was generated and correlated using the described methods. An example elbow plot for the repeated clustering is shown in Fig. 4. It clearly demonstrates that the “elbow” criterion occurs at three clusters, which corresponds to the optimal number of clusters within the data. This was repeated over the 24 STN volumes. The majority (58%) had an optimal cluster number of three (17% = 2, 13% = 4, 8% = 5, 4% = 8). This agrees with animal data in which three functional sub-territories of the STN are well described. Each of the sub-clusters (anterior, middle and posterior), were reliably identified bilaterally in all subjects. The sub-segmented image (Fig. 5) was produced by defining regions present in > 25% at a group level, and in the resulting image each voxel was labelled according to maximal probability. These images correspond well to the human projection of monkey data shown in Yelnik et al. (2007).

### Division of functional zones

**Fig. 6:**
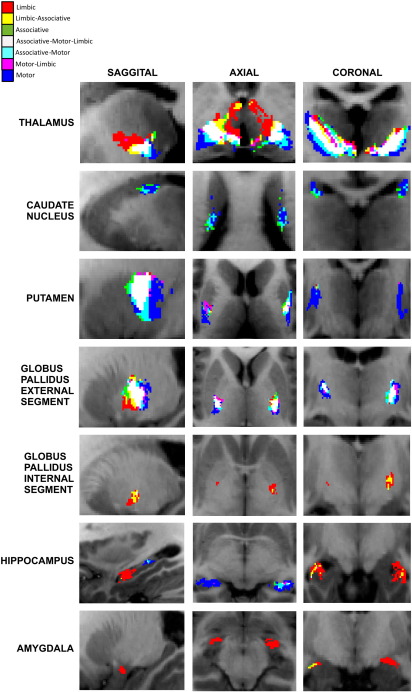
Overlap of group averaged projections from sub-segmented STN regions. Overlap regions are defined by group averaged tractography distributions in standard space for each STN subregion, and then classifying each ROI brain voxel according to the combination of these average distributions that is connected with it. This is summarised in the top left legend. These demonstrate that the associative regions previously shown (Supplementary Material 2) represent an overlapping network between distinctive motor and limbic networks, sharing regions common to both.

Cortical and subcortical connectivity results were recalculated at an individual subject level as previously described. Two methods were used to analyse the sub-segmented regions. First, the subcortical regions were hard-segmented into functional zones based on maximal probability of connection to the corresponding STN cluster. These results are shown in the Supplementary Material 3. Second, overlap with the group-averaged regions was examined as shown in Fig. 6.

#### Subcortical

STN regions separated into two distinctive networks corresponding to the anterior and posterior clusters. The anterior network had unique clusters in the baso-lateral nucleus of the amygdala, anterior hippocampi, posterior-medial GPi, mid GPe and anterior thalamic nuclei. These regions support the hypothesis that the anterior STN is predominately a limbic structure (Kogan and Richter-Levin, 2008; Smith et al., 2004; Yelnik et al., 2007) in agreement with the animal literature (Hamani et al., 2004). Limbic connections were also found in the ventro-lateral thalamus which is a motor region, however this may represent a limbic-motor interface and will be considered further in the discussion. Smaller, bilateral clusters were also present in the mid-putamen region. The cluster size within the anterior “limbic” network was asymmetric, with larger clusters present (at 25% threshold) on the left particularly in the GPi (left — 187 mm^3^ vs right — 10 mm^3^), GPe (843 mm^3^ vs 453 mm^3^) and the amygdala (158 mm^3^ vs 82 mm^3^). Though direct amygdalo-STN connectivity has not been previously demonstrated, a portion of the ventral amygdalofugal pathway passes around and through the STN without terminating (Price and Amaral, 1981), which would account for the observed result. Additionally the parasubthalamic nucleus receives both afferents and efferents from the amygdala (Goto and Swanson, 2004), however a homologous structure has not yet been shown to exist in primates.

Conversely, the posterior network had large, distinctive clusters in the posterior third of the putamen and GPe, mid tail of the caudate nucleus, posterior end of the hippocampi and the ventrolateral nuclei of the thalamus. These regions have been previously defined as motor regions (Draganski et al., 2008; McFarland and Haber, 2000; Yelnik et al., 2007) and support the hypothesis that the posterior STN belongs to a motor network. The remaining central “associative” cluster shared common features with both limbic and motor networks. Analysis of the overlap regions highlights this, showing a gradient from purely limbic regions to purely motor regions with the defined associative network being found between these in the GPe, putamen and thalamus (Fig. 6 and Supplementary Material 5). Additionally, structures receiving purely limbic projections (anterior hippocampus, amygdala and GPi) also have small contributions from the associative projections, as do purely motor regions (posterior putamen, caudate nucleus and posterior hippocampus).

#### Cortical

Most cortical regions possessed some projections to all the STN sub-zones. One region where distinct separation was found was the insula bilaterally, with a predominately motor zone found posteriorly and limbic zone found anteriorly, again more prominently on the left. To disentangle the regional STN cortical connectivity within cortical regions, each voxel was assigned to one of the seven overlap classes. Each region was then described according to the proportion of each type of voxel classification within that area (see Fig. 6 legend). The majority of connecting cortical regions were found to receive contributions from all STN subregions, but with different proportions of each type (summarised in Supplementary Material 4). In most regions the dominant connection is motor and motor-associative. Bilateral symmetrical distributions of sub-region projections appear to be present in few cortical regions. The most obvious exceptions are the supramarginal gyrus and superior frontal sulcus. The limbic connections are more prominent in the left hemisphere except for the middle-frontal gyrus, middle anterior cingulate and superior precentral gyrus where there is right-sided dominance.

## Discussion

Animal studies have demonstrated that the STN projects to a broad array of sub-cortical (Hazrati and Parent, 1992; Kita and Kitai, 1987; Nauta and Cole, 1978) and cortical (Afsharpour, 1985; Degos et al., 2008; Jackson and Crossman, 1981b) targets. These show strong concordance with the current results. Histopathological primate studies (Karachi et al., 2005) have demonstrated STN subdivision into three functional zones, named motor, limbic and associative due to their overlap with the corresponding regions in other striatal structures (Karachi et al., 2005). Presented data based on non-invasive anatomical connectivity supports this functional subdivision in humans, and begins to elucidate the relationships between these regions.

### Motor STN

The posterior aspect of the STN demonstrates connectivity with targets consistent with a motor structure. Namely, these are the posterior insula (Chikama et al., 1997; Flynn et al., 1999; Afif et al., 2010), posterior putamen and GPe, mid-caudate nucleus and ventro-lateral thalamic nuclei (Yelnik et al., 2007). Projections to the posterior GPi were expected (Yelnik et al., 2007) but not found, but instead were present in the associative and limbic portions of the STN. This may be due to several factors, including misclassification of the posterior GPi during manual segmentation, atlas differences (Morel, 2007) or due to the anatomical arrangement of fibres. Some STN fibres exit via its anterior-medial pole to reach GPi via ansa lenticularis (Marani et al., 2008), whilst fibres of the subthalamic fasciculus reach the GPi by crossing the internal capsule at right angles (Parent and Parent, 2004). Both of these factors could contribute to produce the observed tractography result. Pulse gating, higher angular resolution, or a higher b value may help resolve this (Behrens et al., 2007), especially if sub-population sampling was used during the tractography.

**Fig. 7:**
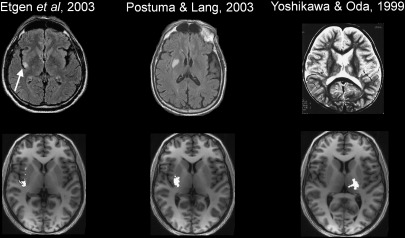
Overlap between published isolated lesions causing non-STN hemiballismus (top row) and motor-STN projections defined in the current study (bottom row). Top row images reproduced with kind permission from Springer Science and Business Media (left) and Elsevier (centre and right) and with the corresponding authors' permission.

Injury to the STN produces the rare clinical symptom of hemiballismus, an involuntary, irregular flailing movement affecting the contralateral limbs (Shannon 2005). STN ablation in primates reproduces this symptom (Carpenter, 1955). However, it has also been described following localised damage to other regions. Case reports detailing non-STN hemiballismus demonstrate focal lesions affecting the posterior insula (Etgen et al., 2003), posterior putamen, posterior GPe (Posturma and Lang, 2003), and ventrolateral thalamus (Schmahmann, 2003; Yoshikawa and Oda, 1999). These lesions overlap closely with motor STN projections (Fig. 7). This supports the hypothesis that hemiballismus arises through interruption of STN afferent and efferent projections, causing an imbalance between inhibitory and excitatory transmissions that modulate normal motor control (Bhatia and Marsden, 1994).

### Limbic STN

The anterior STN was found to project to the baso-lateral amygdala, inferio-mid putamen, mid-GPe and ventral-anterior thalamus. These projections are consistent with a limbic structure (Kogan and Richter-Levin, 2008; Smith et al., 2004). Evidence exists from patients undergoing STN deep brain stimulation that further supports this possibility. Mallet et al. (2007) report two cases of reproducible hypomania arising from anterior STN stimulation. The acute effects of limbic STN stimulation include spontaneous laughter and pathological crying (Krack et al., 2001; Wojtecki et al., 2007). These are characterised by marked motor responses and may explain why limbic projections were also found in the ventro-lateral thalamus. Other limbic DBS effects such as elevated mood, cognitive and perceptual changes manifest after prolonged (> 12 h) STN stimulation (Herzog et al., 2003; Mallet et al., 2007; Mandat et al., 2006; Temel et al., 2005, 2006). This suggests that human STN DBS may modulate neural function by different methods including both short and long term mechanisms of neuroplasticity (Destexhe and Marder, 2004).

### Associative STN

There is sparse literature concerning the associative STN. The projections found in this study are consistent with the definition provided by Hamani et al. (2004). Further, the current study demonstrated that the associative region projects to both limbic and motor pathways, providing a link between two distinct circuits, which may represent a functional gradient rather than distinct sub-regions with clear cytoarchitectual boundaries (Karachi et al., 2005). Indeed, the STN connections with the putamen, thalamus and GPe demonstrate a distinctive functional topological gradient, which provides a morphological correlate with the closed reciprocal, open non-reciprocal spiral loops previously demonstrated in other parts of the basal ganglia (Haber, 2003; Haber et al., 2000) (Supplementary Material 5).

### Topological functional arrangement of the STN

In this study, we have described the topological properties of STN subdivisions and assigned them to a functional network based on the patterns of connectivity. While this result is consistent with previous descriptions (Joel and Weiner, 1997; Yelnik et al., 2007), it is also the case that the STN connectivity patterns are complex. There are different patterns between the efferent and afferent projections (Joel and Weiner, 1997), in addition to several overlapping somatotopic representations to cortical and subcortical regions (Joel and Weiner, 1997; Nambu et al., 1996; Miyachi et al., 2006; Romanelli et al., 2005). The current method used to parcellate the STN is based on quantifying differences between tractography distributions, which represent the sum total of afferents and efferents from a given voxel to all regions (given the lack of information in DWI data regarding anatomical polarity). The more divergent the paths, the greater the measured differences will be. This means subtle, overlapping, region dependent somatotopy may well require either a much higher level of clustering to detect or alternatively, a region selective analysis rather than the global approach currently used. Our results bring strong evidence that the somatotopic differences between the STN and other subcortical regions that drive this segmentation. These are most similar to the previously described pattern of afferent projections (Joel and Weiner, 1997). Additionally, though we found evidence of a spatial-gradient of connections particularly between the thalamus, GPe and putamen, the presence of STN sub-region specific projections both within these regions and also to the insula, caudate, amygdala and hippocampus, indicates that specialised, closed networks do exist. It may be the case that there are unique limbic and motor STN zones, and that the associative zone represents an overlapping, somatotopically arranged transition between the two. This would provide an anatomical substrate for communication between two distinctive closed networks similar to the spiral loops that have previously been described for nigro-striatal pathways (Haber, 2003; Haber et al., 2000).

### STN-hippocampal connectivity

The STN-hippocampal connectivity reported in this study is unprecedented, which may be due to a combination of reasons. We observed well-circumscribed clusters that could be missed in lesion studies, a previously noted problem in the hippocampus (Chronister et al., 1975; Sikes et al., 1977). Afferents to the hippocampus have been reported from the nearby zona incerta, ventral tegmental area, substantia nigra, pedunculopontine nucleus (PPN) (Amaral and Cowan, 1980), parasubthalamic nucleus (Goto and Swanson, 2004) and anterior thalamic nuclei (Von Gudden, 1881). Possibly, projection fibres from these regions are closely associated with the STN (Jackson and Crossman, 1981b) and for this reason were included in the tractography results presented here. Acknowledging these potential confounders, the results remain noteworthy. Using blind parcellation of the STN with no prior specification of “limbic” or “motor” structures, we have shown connectivity to other regions consistent with these divisions. Likewise, the hippocampi show a clear sub-division into an anterior limbic/limbic associative region, and a posterior motor/motor-associative region. This parcellation of the hippocampus has anatomical validity. Primates and rodent studies demonstrate preferential posterior hippocampus involvement in spatial tasks (Colombo et al., 1998; Moser and Moser, 1998; Moser et al., 1995), a finding replicated in fMRI human studies (Burgess et al., 2002; Maguire et al., 2000). The MNI coordinates within the centre of the motor-STN hippocampal clusters ([31,-22,10],[-31,-28,-7]) are precisely where volumetric grey matter changes were demonstrated in experienced London Taxi drivers (Maguire et al., 2000). A recent review (Fanselow and Dong, 2010) proposed that ample evidence exists for dividing the hippocampus into distinct zones; a posterio-superior “cold” zone for locomotion and navigation; and anterior–inferior “hot” zone for motivated and emotive behaviours. Although too early to speculate whether the STN plays a role in hippocampal processing, these findings do support an anterior–posterior functional split within the hippocampus.

### Comparison with previous studies

One previous study that formally examined tractography from the STN was by Aravamuthan et al. (2007). However, there are several methodological differences that make direct comparison difficult. Instead of examining global STN connectivity, they instead examined for connections that were strongly implicated in STN connectivity, namely the motor and pre-motor cortices. In this regard our results are consistent with their findings, and build upon them by examining all cortical and sub-cortical regions. Additionally, they specifically examined for motor somatotopy, which we cannot comment on in this current study. However, the most important distinction is the selection of seed voxels. In this current study, considerable effort was taken to directly visualise and define all the voxels falling within the STN. In contrast, Aravamuthan et al. defined a single voxel based on surrounding landmarks that they were confident fell within the STN. The presence of cerebellar connections, which were absent in this study and second order connections via the pontine nuclei (Bostan et al., 2010), indicate that perhaps some of their STN seeds additionally sampled the nearby fasciculus cerebellothalamicus (Aström et al., 2010; Gallay et al., 2008).

### Surgical implications

STN DBS is increasingly used in the treatment of movement disorders, predominately for the motor symptoms of Parkinson's disease. The sensorimotor region of the STN has been reported as the optimal stimulation site (Lanotte et al., 2002; Romanelli et al., 2005; Voges et al., 2002). However, STN DBS is not infrequently associated with adverse events, predominately cognitive and neuropsychiatric complications, speech problems and balance disturbances (Hariz et al., 2008). Whilst these could represent advancing Parkinson's disease, it is thought that a proportion result from stimulation of unwanted fibre pathways, e.g. speech disturbance due to stimulation of the cerbellothalamic fasciculus (Aström et al., 2010; Tripoliti et al., 2008).

The current study provides evidence that the human STN can be segmented on neuroimaging in vivo into its three functional zones. This segmentation was demonstrated in individual subjects (Supplementary Material 6) as well as across subjects. Replication in patients with basal ganglia pathology may provide prognostic information with regard to response to STN DBS. Additionally, such connectivity maps could conceivably play a role in image-based targeting of the motor portion of the STN, particularly when combined with T2* sequences optimised specifically for visualising the STN to make identification and delineation of the nucleus more accurate.

### Methodological limitations and considerations

This study is not without limitations, which must be taken into consideration when interpreting the results. First, probabilistic tractography is, as the name implies, a probabilistic method. Seed and target region size, distance of tracking, regions with dense crossing fibres, MRI artefact and noise will all affect the absolute PICo value obtained and therefore certain regions that may be expected could fall below the threshold and not be seen, or only occur in a few individuals. For this reason, we report both “weak” and “strong” connectivity values. Second, it is not possible to determine the direction of connections using DT, rather they represent the sum total of both afferents and efferents. As such, it is not possible to place the current framework in terms of direct, indirect and hyperdirect loops. Instead, this paper attempts to describe the spatial arrangement of the network between subcortical structures and the STN, showing similar results to anatomical studies and supporting the concept of spiral loops (Supplementary Material 4). Third, tractography relies on tracking groups of axons by virtue of their anisotropy, hence disynaptic connections via intermediary grey matter structures, such as those to the cerebellum via the PPN (Bostan et al., 2010), are unlikely to be seen. Additionally, this study visually assessed for the “elbow point” which is a common approach (Young et al., 2006), however it would be better to develop an objective measure to detect this based on the rate of gradient descent. Finally identification of the STN relies on the MRI contrast provided by the neuromelanin. It is known that the iron content falls in the posteromedial aspect (Dormont et al., 2004), and therefore this area may not be visible on R2* weighted scans. However, our absolute STN volumes fall well within the range of the previous studies (Dormont et al., 2004; Hardman et al., 2002; Lange et al., 1976; Lévesque and Parent, 2005). Additionally, this study demonstrates high concordance with previous animal studies, and also identifies limbic, associative and motor networks based purely on their connections and the literature. Though it is possible that the motor portion was underestimated, these results suggest that three regions were reliably identified. Though higher field strengths may help resolve this further, the additional advantage with this study is it was performed at a clinically relevant, widely used MRI field strength and as such has the potential to be utilised both in a hospital setting and by the broader research community. One future improvement will be to use T2* weighted sequences specifically optimised for imaging the STN, improving structural identification of the most superior sensorimotor region.

## Conclusion

In summary, this DWI study demonstrated the existence of three distinct sub-regions within the human STN and provided detailed analysis of both whole and sub-regional STN connectivity to cortical and sub-cortical structures in vivo. Using connectivity data, we have provided evidence that supports an anterior “limbic”, middle “associative” and posterior “motor” STN existing in humans. Anatomical precision was achieved by employing an automated segmentation approach for the majority of ROIs. The current gold standard for STN localization is direct manual identification and was employed in this study. These findings are novel in several aspects: First, the use of a data driven method to independently determine optimal cluster number within a region of interest, thereby confirming the existence of three distinct regions within the STN. Second, STN was segmented into functional sub-regions with corresponding cortical connectivity in vivo.

Our motivation for studying the STN was to provide a methodological framework with which to study both pathological processes affecting this network, and also surgical consequences of deep brain stimulator surgery. Conformation and replication of previous findings in non-human primates provide strong supportive evidence for these results, and substantiates the proposed framework.

The following are the supplementary materials related to this article.

**Supplementary Material 1:**
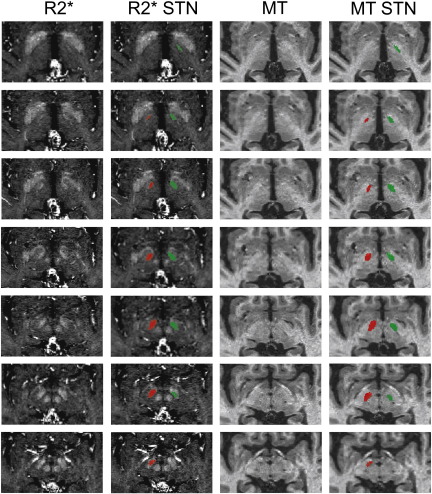
Delineation of an STN shown as the hyperintense region on the R2* images (left — green, right — red), progressing from the most superior tip (top) to inferior aspect (bottom) in 1 mm slices. The corresponding MT image is also shown, which helped identify the surrounding boundaries by using ITK-SNAP multisession view (STN is not visible on these images). The following boundaries were used in addition to direct visualisation on the R2* images: Inferiorly — The mid-point of the red nucleus, superior aspect of the optic tract and substantia nigra; Anteriorly — The posterolateral wall of the hypothalamus, defined as the grey matter between the mamillothalamic tract posteriorly and fornix anteriorly; Medially — The white matter situated between the thalamus and red nucleus; Laterally — The internal capsule; Superiorly — The anterior commissure.

Supplementary Material 2 STN literature review. A summary table of the proportions of connections reported and the papers used for review are listed.

**Supplementary Material 3:**
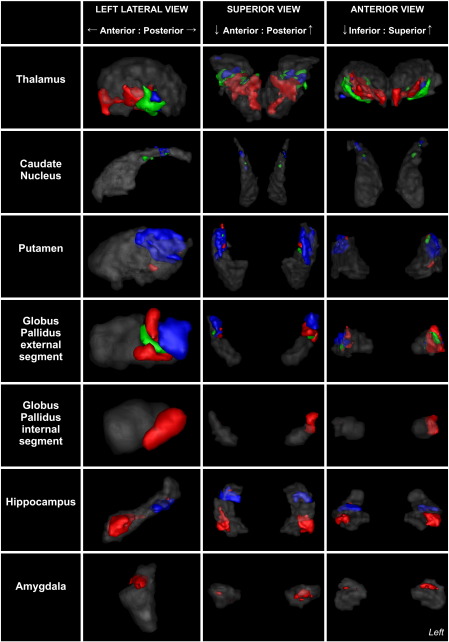
Renderings displaying the spatial distributions of sub-cortical connectivity from the ipsilateral sub-segmented STN. Regions were identified as areas where the corresponding tractography overlapped in 3 or more subjects at a tractography threshold of 1%. The individual voxel values of the groups were defined according to the STN sub-region they were maximally assigned to across all subjects. Colour coded according to the calculated STN regions shown previously (red = anterior “Limbic” STN, green = middle “associative” STN, blue = posterior “motor” STN). Outline of the region of interest shown in transparent grey. Substantia nigra and red nucleus not shown as these areas were strongly connected to all regions.

**Supplementary Material 4:**
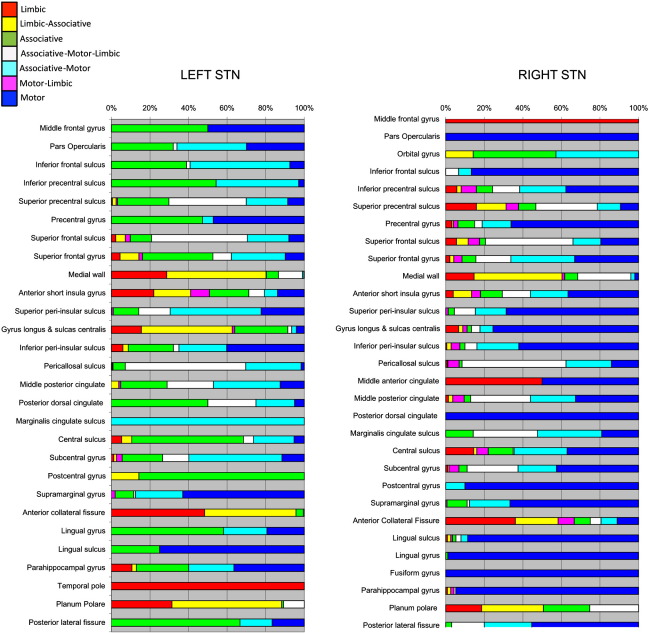
Regional ipsilateral cortical connectivity from sub-segmented STN. Regions with connectivity present in ≥ 25% of subjects shown (see Table 1). Bars represent the proportion of all connected voxels within a cortical region that are connected to a corresponding STN zone (limbic, motor or associative). Overlap regions are shown, defined as a voxel that meets threshold for two or more STN sub-distributions.

**Supplementary Material 5:**
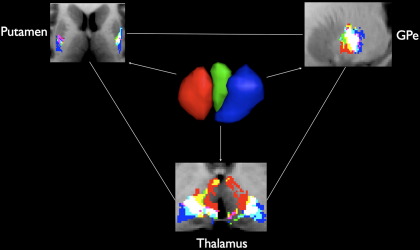
GPe–putamen–thalamic relay ring showing unique functional topological gradient, defined as projections to all STN-sub regions respecting a somatotopic gradient. These were only present in these areas, and provide a morphological correlate for closed reciprocal, open non-reciprocal spiral loops.

**Supplementary Material 6:**
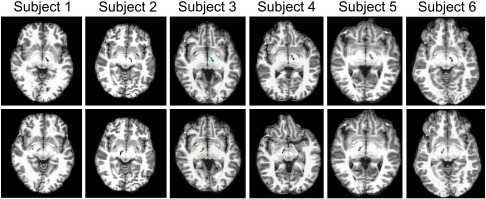
STN segmentations from 6 individuals.

## Figures and Tables

**Table 1:** Imaging parameters.

Image type	Slice no	FOV (mm^2^)	Acquisition matrix (voxels)	TR (ms)	TE (ms)	Flip angle	Echo no.	Notes
MDEFT	176	224 × 256	224 × 256	20.66	8.42	25	–	Resolution = 1 mm^3^ T1 = 640 ms Bandwidth = 178 Hz/pixel Acquisition time = 12 min
DTI	80	220 × 220	128 × 128	170	102	90;180	–	Resolution = 1.7 mm^3^ Average of two acquisitions Acquisition time = 32 min 68 images: 61 evenly distributed directions (b = 1000 s/mm^2^), (Jansons and Alexander, 2003) 7 b = 100 s/mm^2^ images
MTw	176	240 × 256	240 × 256	23.7	[2.2:2.5:14.7]	6	6	Resolution = 1 mm^3^ Parallel imaging (GRAPPA) (Griswold et al., 2002) along phase encoding direction Partition partial Fourier (6/8) Bandwidth = 425 Hz/pixel
T1w	176	240 × 256	240 × 256	23.7	[2.2:2.5:14.7]	20	8
PDw	176	240 × 256	240 × 256	18.7	[2.2:2.5:19.7]	6	6
B1-Map	48	192 × 256	48 × 64	500	(SE:37.06; STE:68.26)	SE:[230: − 10:130]	2
Fieldmap	64	192 × 192	64 × 64	1020	10; 12.46	90	2

**Table 2:** Summary of group STN ipsilateral connectivity. Results ≥ 25% reported (i.e. connections present in 3 or more subjects).

	Percentage of subjects	
Left	Right
*Ipsilateral sub-cortical regions:*		
Thalamus	100	100
Caudate nucleus	100	100
Putamen	100	100
Globus pallidus external segment	100	100
Globus pallidus internal segment	100	83
Substantia nigra	100	100
Red nucleus	100	100
Hippocampus	83	75
Amygdala	67	58

*Ipsilateral cortical regions:*		
Frontal		
Frontal middle gyrus	25	33
Pars opercularis	58	25
Orbital gyrus	–	33
Inferior frontal sulcus	50	33
Inferior precentral sulcus	50	58
Superior precentral sulcus	75	58
Precentral gyrus	33	50
Superior frontal sulcus	67	67
Superior frontal gyrus	50	67
Medial wall	83	83
Insula		
Anterior short insula gyrus	58	58
Superior peri-insular sulcus	75	58
Gyrus longus and sulcus centralis	75	58
Inferior peri-insular sulcus	67	92
Cingulate		
Pericallosal sulcus	92	100
Middle anterior cingulate	25	42
Middle posterior cingulate	75	92
Posterior dorsal cingulate	50	33
Marginalis cingulate sulcus	42	50
Parietal		
Central sulcus	42	50
Subcentral gyrus	75	75
Postcentral gyrus	33	42
Supramarginal gyrus	58	42
Anterior collateral fissure	67	42
Temporal		
Lingual gyrus	50	58
Lingual sulcus	33	42
Fusiform gyrus	25	33
Parahippocampal gyrus	58	58
Temporal pole	33	–
Planum polare	67	58
Posterior lateral fissure	50	58
