# Supplementary material for: Confirmation of functional zones within the human subthalamic nucleus: Patterns of connectivity and sub-parcellation using diffusion weighted imaging
Source: Neuroimage. 2012 Mar;60(1):83–94. doi: 10.1016/j.neuroimage.2011.11.082 (PMC3315017; doi:10.1016/j.neuroimage.2011.11.082)
Supplement: Supplementary Material 2 — STN literature review. A summary table of the proportions of connections reported and the papers used for review are listed. [file mmc1.doc]

**Supplementary Material 2**

|  |  | **STN** | | | **Para-STN** | | | **STN** | | **Para-STN** | |
| --- | --- | --- | --- | --- | --- | --- | --- | --- | --- | --- | --- |
| **Subcortical** | **Region** | **N Afferent** | **N Efferent** | **N Both** | **N Afferent** | **N Efferent** | **N both** | **Afferent (%)** | **Efferent (%)** | **Afferent (%)** | **Efferent (%)** |
|  | Pallidum (unspecified) | 3 | 1 | 0 | 0 | 0 | 0 | 1.28 | 0.54 | 0.00 | 0.00 |
|  | Dorsal Pallidum | 2 | 0 | 0 | 0 | 0 | 0 | 0.85 | 0.00 | 0.00 | 0.00 |
|  | Ventral Pallidum | 5 | 2 | 1 | 0 | 0 | 0 | 2.55 | 1.63 | 0.00 | 0.00 |
|  | Gpi | 6 | 25 | 5 | 0 | 0 | 0 | 4.68 | 16.30 | 0.00 | 0.00 |
|  | Gpe | 11 | 21 | 17 | 0 | 0 | 0 | 11.91 | 20.65 | 0.00 | 0.00 |
|  | Putamen | 1 | 8 | 1 | 0 | 0 | 0 | 0.85 | 4.89 | 0.00 | 0.00 |
|  | Nucleus Accumbens (core) | 2 | 0 | 0 | 0 | 0 | 0 | 0.85 | 0.00 | 0.00 | 0.00 |
|  | Caudate Nucleus | 1 | 5 | 2 | 0 | 0 | 0 | 1.28 | 3.80 | 0.00 | 0.00 |
|  | Striatum (unspecified) | 1 | 5 | 1 | 0 | 0 | 1 | 0.85 | 3.26 | 0.43 | 0.54 |
|  | Substantia Innominata | 1 | 3 | 0 | 0 | 0 | 0 | 0.43 | 1.63 | 0.00 | 0.00 |
|  | Substantia Nigra | 5 | 21 | 7 | 0 | 0 | 0 | 5.11 | 15.22 | 0.00 | 0.00 |
|  | Ventral Tegmental Area | 0 | 1 | 0 | 0 | 0 | 0 | 0.00 | 0.54 | 0.00 | 0.00 |
|  | Bed Nucleus of Stria Terminalis | 4 | 0 | 0 | 0 | 0 | 1 | 1.70 | 0.00 | 0.43 | 0.54 |
|  | Amygdala | 1 | 0 | 0 | 0 | 0 | 1 | 0.43 | 0.00 | 0.43 | 0.54 |
|  | Hippocampus | 0 | 0 | 0 | 0 | 1 | 0 | 0.00 | 0.00 | 0.00 | 0.54 |
|  | Hypothalamus | 5 | 2 | 0 | 0 | 0 | 1 | 2.13 | 1.09 | 0.43 | 0.54 |
| **Thalamus** | Thalamus (Unspecified) | 0 | 0 | 0 | 0 | 0 | 0 | 0.00 | 0.00 | 0.00 | 0.00 |
|  | Ventrolateral Thalamus | 0 | 3 | 0 | 0 | 0 | 0 | 0.00 | 1.63 | 0.00 | 0.00 |
|  | Ventroanterior Thalamus | 0 | 2 | 0 | 0 | 0 | 0 | 0.00 | 1.09 | 0.00 | 0.00 |
|  | Centromedial Thalamus | 8 | 1 | 1 | 0 | 1 | 0 | 3.83 | 1.09 | 0.00 | 0.54 |
|  | Mediodorsal Thalamus | 0 | 1 | 0 | 0 | 1 | 0 | 0.00 | 0.54 | 0.00 | 0.54 |
|  | Paraventricular Thalamus | 0 | 0 | 0 | 0 | 1 | 0 | 0.00 | 0.00 | 0.00 | 0.54 |
|  | Parafascicular Thalamus | 15 | 1 | 0 | 0 | 0 | 0 | 6.38 | 0.54 | 0.00 | 0.00 |
|  | Nucleus Reticularis Thalami | 0 | 1 | 0 | 0 | 0 | 0 | 0.00 | 0.54 | 0.00 | 0.00 |
|  | Posterior Intralaminar Nuclei | 1 | 0 | 0 | 0 | 0 | 0 | 0.43 | 0.00 | 0.00 | 0.00 |
|  | Zona Incerta | 1 | 2 | 0 | 0 | 0 | 0 | 0.43 | 1.09 | 0.00 | 0.00 |
| **Cortex** | Frontal Lobe (unspecified) | 2 | 1 | 0 | 0 | 0 | 0 | 0.85 | 0.54 | 0.00 | 0.00 |
|  | Temporal Lobe (unspeified) | 0 | 1 | 0 | 0 | 0 | 0 | 0.00 | 0.54 | 0.00 | 0.00 |
|  | Prefrontal Region | 2 | 1 | 0 | 0 | 0 | 0 | 0.85 | 0.54 | 0.00 | 0.00 |
|  | Medial Prefrontal Region | 3 | 1 | 1 | 0 | 0 | 0 | 1.70 | 1.09 | 0.00 | 0.00 |
|  | Dorsolateral prefrontal cortex | 4 | 0 | 1 | 0 | 0 | 0 | 2.13 | 0.54 | 0.00 | 0.00 |
|  | Insular (Agranular) | 1 | 0 | 0 | 0 | 1 | 0 | 0.43 | 0.00 | 0.00 | 0.54 |
|  | Insula (Granular) | 2 | 0 | 0 | 0 | 0 | 0 | 0.85 | 0.00 | 0.00 | 0.00 |
|  | Infralimbic area | 0 | 0 | 0 | 0 | 1 | 0 | 0.00 | 0.00 | 0.00 | 0.54 |
|  | Enterhinal Cortex | 0 | 0 | 0 | 0 | 1 | 0 | 0.00 | 0.00 | 0.00 | 0.54 |
|  | Anterior Cingulate | 4 | 0 | 0 | 0 | 0 | 0 | 1.70 | 0.00 | 0.00 | 0.00 |
|  | Medial Cingulate | 3 | 0 | 0 | 0 | 0 | 0 | 1.28 | 0.00 | 0.00 | 0.00 |
|  | Cingulate motor areas | 4 | 0 | 0 | 0 | 0 | 0 | 1.70 | 0.00 | 0.00 | 0.00 |
|  | Frontal Eye Field | 7 | 1 | 0 | 0 | 0 | 0 | 2.98 | 0.54 | 0.00 | 0.00 |
|  | Supplementary Eye Field | 2 | 0 | 0 | 0 | 0 | 0 | 0.85 | 0.00 | 0.00 | 0.00 |
|  | Pre-SMA | 8 | 0 | 0 | 0 | 0 | 0 | 3.40 | 0.00 | 0.00 | 0.00 |
|  | SMA | 11 | 0 | 0 | 0 | 0 | 0 | 4.68 | 0.00 | 0.00 | 0.00 |
|  | Pre-Motor Area | 8 | 0 | 0 | 0 | 0 | 0 | 3.40 | 0.00 | 0.00 | 0.00 |
|  | Primary Motor | 15 | 3 | 0 | 0 | 0 | 0 | 6.38 | 1.63 | 0.00 | 0.00 |
|  | Primary Sensory | 6 | 1 | 0 | 0 | 0 | 0 | 2.55 | 0.54 | 0.00 | 0.00 |
| **Mesencephalon** | Pendunculopontine Nucleus | 13 | 8 | 4 | 0 | 0 | 0 | 7.23 | 6.52 | 0.00 | 0.00 |
|  | Mesopontine Tegmentum | 1 | 1 | 0 | 0 | 0 | 0 | 0.43 | 0.54 | 0.00 | 0.00 |
|  | Laterodorsal Tegmental Nucleus | 4 | 1 | 0 | 0 | 0 | 0 | 1.70 | 0.54 | 0.00 | 0.00 |
|  | Midbrain Extrapyramidal Area | 1 | 2 | 0 | 0 | 0 | 0 | 0.43 | 1.09 | 0.00 | 0.00 |
|  | Superior Colliculus | 2 | 0 | 0 | 0 | 0 | 0 | 0.85 | 0.00 | 0.00 | 0.00 |
|  | Raphe Nucleus (Dorsal) | 3 | 1 | 1 | 0 | 0 | 0 | 1.70 | 1.09 | 0.00 | 0.00 |
|  | PAG | 0 | 3 | 0 | 0 | 0 | 0 | 0.00 | 1.63 | 0.00 | 0.00 |
|  | Cuneiform Nucleus | 1 | 0 | 0 | 0 | 0 | 0 | 0.43 | 0.00 | 0.00 | 0.00 |
| **Pons** | Pontine Nuclei | 4 | 2 | 0 | 0 | 0 | 0 | 1.70 | 1.09 | 0.00 | 0.00 |
|  | Motor Trigeminal Nucleus | 0 | 0 | 0 | 0 | 1 | 0 | 0.00 | 0.00 | 0.00 | 0.54 |
|  | Parabrachial Nucleus | 2 | 0 | 0 | 0 | 1 | 0 | 0.85 | 0.00 | 0.00 | 0.54 |
|  | Cerebellum | 0 | 0 | 0 | 0 | 0 | 0 | 0.00 | 0.00 | 0.00 | 0.00 |
|  | Locus Coeruleus | 2 | 0 | 0 | 0 | 0 | 0 | 0.85 | 0.00 | 0.00 | 0.00 |
| **Medulla** | Ventral Respiratory Group | 0 | 1 | 0 | 0 | 0 | 0 | 0.00 | 0.54 | 0.00 | 0.00 |
|  | Nucleus of Solitary Tract | 0 | 0 | 0 | 0 | 1 | 0 | 0.00 | 0.00 | 0.00 | 0.54 |
| **Spine** | Spinal Cord (cervical) | 0 | 4 | 0 | 0 | 0 | 0 | 0.00 | 2.17 | 0.00 | 0.00 |

Supplementary table 1: Absolute numbers (N) of articles reporting a particular connection, and the proportion (%) of all connections reported this represents (as detailed in the methods).

**Articles Reviewed**

33 Dong et al 2000

34 Sato et al 2000a

35 Inase et al 1999

36 Gaytán & Pásaro 1998

37 Smith et al 1998

38 Joel & Weiner 1997

39 Shink et al 1996

40 Nambu et al 1996

41 Deschênes et al 1996

42 Zahm et al 1996

43 Bevan & Bolam 1995

44 Bell et al 1995

45 Parent & Hazrati 1995

46 Lavoie & Parent 1994

47 Smith et al 1994

48 Féger et al 1994

49 Tokuno et al 1994

50 Bevan et al 1994

51 Kita, & Kitai 1994

52 Groenewegen et al 1993

53 Spooren et al 1993

54 Noda & Oka 1993

55 Haber et al 1993

56 Parent & Hazrati 1993

57 Rouiller et al 1993

58 Hazrati, & Parent 1992b

59 Semba, & Fibiger 1992

60 Steininger et al 1992

61 Hazrati, & Parent 1992a

62 Sadikot et al 1992

63 Moriizumi, & Hattori 1992

64 Nakano et al 1990

65 Groenewegen, & Berendse 1990

1 Mathai & Smith 2011

2 Nambu 2011

3 Tripathi et al 2010

4 Ciesielska et al 2011

5 Rico et al 2010

6 Bostan et al 2010

7 Cebrián, & Prensa 2010

8 Mascaro et al 2009*

9 Gradinaru et al 2009

10 Parent, & Parent 2007

11 Sadikot, & Rymar 2009

12 Benabid 2009

13 Marani et al 2008

14 Degos et al 2008

15 Kita 2007

16 Nambu 2007

17 Bevan et al 2007

18 Miyachi et al 2006

19 Winn 2006

20 Tandé et al 2006

21 Temel et al 2005

22 Wood, & Swann 2005

23 Romanelli et al 2005

24 Karachi et al 2005

25­ Goto & Swanson 2004*

26 Kelly & Strick 2004

27 Nambu et al 2002

28 Takada et al 2001

29 Prensa, & Parent 2001

30 Baron et al 2001

31 Nakano et al 2000

32 Sato et al 2000b

66 Canteras et al 1990

100 Beckstead 1983

101 Sloper et al 1983

102 Sugimoto & Hattori 1983

103 Hammond et al 1983

104 Sugimoto et al 1983

105 Saper & Loewy 1982

106 Gerfen et al 1982

107 Robertson & Feiner 1982

108 Usunoff et al 1982

109 Henkel 1981

110 Carpenter et al 1981b

111 Carpenter et al 1981a

112 Jackson, & Crossman 1981

113 Ricardo 1980

114 Nomura et al 1980

115 Van Der Kooy & Hattori 1980

116 McBride, & Larsen 1980

117 Van der Kooy et al 1981

118 Romansky et al 1980

119 DeVito et al 1980

120 Monakow et al 1978

121 Deniau et al 1978

122 Nauta, & Cole 1978

123 Kanazawa et al 1976

124 Kim et al 1976

125 Carpenter et al 1976

126 Hattori et al 1975

127 Nakamura, & Sutin 1972

128 Carpenter, & Strominger 1967

129 Whittier, & Mettler 1949

130 Glees & Wall 1946

*parasubthalamic nucleus

67 Smith et al 1990b

68 Smith et al 1990a

69 Beart et al 1990

70 Carpenter & Jayaraman 1990

71 Miyashita & Tamai 1989

72 Parent et al 1989

73 Zahm 1989

74 Lee et al 1988

75 Canteras et al 1988

76 Mizuno et al 1988

77 Stanton et al 1988

78 Takada et al 1988

79 Parent & Smith 1987

80 Takada et al 1987

81 Moriizumi et al 1987

82 Leichnetz et al 1987

83 Scarnati et al 1987

84 Veening et al 1987

85 Kita & Kitai 1987

86 Swanson et al 1987

87 Miyata 1986

88 Takada et al 1986

89 Woolf & Butcher 1986

90 Velayos & Reinoso-Suárez 1985

91 Afsharpour 1985

92 Haber et al 1985

93 Royce & Mourey 1985

94 Chiba & Murata 1985

95 Steeves & Jordan 1984

96 Royce & Laine 1984

97 Jürgens 1984

98 Sugimoto & Hattori 1984

99 Parent & De Bellefeuille 1983

|  | **REGION** | **References** |
| --- | --- | --- |
| Subcortical | Pallidum (unspecified) | 13,89,109,127 |
|  | Dorsal Pallidum | 67,93 |
|  | Ventral Pallidum | 1,39,45,46,53,66,67,93 |
|  | Gpi | 2,10,15,17,21,24,25,28,31,32,33,38,39,40,43,46,48,54,57,59,62,66,68,69,70,72,82,86,111,112,114  116,122,123,129 |
|  | Gpe | 2,10,13,14,16,17,21,24,28,32,33,35,38,39,40,45,46,52,54,55,56,57,59,62,64,67,68,69,70,72,73,79  80,82,86,111,112,114,116,117,118,119,120,122,123,125,128,129,130 |
|  | Putamen | 13,39,68,70,80,101,123 |
|  | Nucleus Accumbens (core) | 4,43 |
|  | Caudate Nucleus | 13,39,46,68,80,97,101 |
|  | Striatum (unspecified) | 3,10,13,26,33,57,66,79 |
|  | Substantia Innominata | 46,67,68,86 |
|  | Substantia Nigra | 2,3,7,10,13,14,21,28,30,33,38,39,46,51,55,57,66,67,68,70,79,80,86,100,106,107,111,114,116,122  123,124,126 |
|  | Ventral Tegmental Area | 66 |
|  | Bed Nucleus of Stria Terminalis | 22,26,34,46,74 |
|  | Amygdala | 26,46 |
|  | Hippocampus | 26 |
|  | Hypothalamus | 26,46,66,67,85,87,95,116, |
| Thalamus | Ventrolateral Thalamus | 5,123,130 |
|  | Ventroanterior Thalamus | 5,123 |
|  | Centromedial Thalamus | 11,12,21,2,32,46,55,63,70,94,103,105 |
|  | Mediodorsal Thalamus | 26,91 |
|  | Paraventricular Thalamus | 26 |
|  | Parafascicular Thalamus | 11,12,17,20,21,32,46,49,55,63,66,67,70,94,103 |
|  | Nucleus Reticularis Thalami | 13 |
|  | Posterior Intralaminar Nuclei | 42 |
|  | Zona Incerta | 13,46,68 |
| Cortex | Frontal Lobe (unspecified) | 39,55,86 |
|  | Temporal Lobe (unspecified) | 13 |
|  | Prefrontal Region | 13,14,46 |
|  | Medial Prefrontal Region | 14,67,83,88,121 |
|  | Dorsolateral prefrontal cortex | 32,39,46,88,121 |
|  | Insular (Agranular) | 13,26, |
|  | Insula (Granular) | 46,67 |
|  | Infralimbic area | 26 |
|  | Entorhinal Cortex | 26 |
|  | Anterior Cingulate | 13,23,46,67 |
|  | Medial Cingulate | 13,46,83 |
|  | Cingulate motor areas | 1,2,23,29 |
|  | Frontal Eye Field | 28,39,46,71,78,83,92,121 |
|  | Supplementary Eye Field | 28,32 |
|  | Pre-SMA | 1,2,13,17,28,32,36,92 |
|  | SMA | 1,2,13,17,23,28,32,36,41,92,98 |
|  | Pre-Motor Area | 23,28,32,39,58,67,92,121 |
|  | M1 | 1,2,9,13,14,17,18,23,27,28,32,39,41,46,58,67,92,102 |
|  | Primary Sensory | 13,14,46,67,76,92,102 |
|  |  |  |
| Mesencephalon | Pendunculopontine Nucleus | 13,19,38,44,46,47,61,67,68,70,74,79,80,82,84,86,90,99,104,111,113,115,123 |
|  | Mesopontine Tegmentum | 44,46 |
|  | Laterodorsal Tegmental Nucleus | 44,46,90,110,130 |
|  | Midbrain Extrapyramidal Area | 44,61,66 |
|  | Superior Colliculus | 13,50 |
|  | Raphe Nucleus (Dorsal) | 13,21,46,67,86 |
|  | PAG | 13,68,69 |
| Pons | Cuneiform Nucleus | 96 |
|  | Pontine Nuclei | 6,17,46,86,108,117 |
|  | Motor Trigeminal Nucleus | 9 |
|  | Parabrachial Nucleus | 26,46,74 |
|  | Locus Coeruleus | 46,111 |
| Medulla | Ventral Respiratory Group | 37 |
|  | Nucleus of Solitary Tract | 26 |
|  | Spinal Cord (cervical) | 13,38,77,81 |

Supplementary table 2: Summary of literature review

**STN Tract-Tracing Review References**

Afsharpour, S., 1985, Topographical projections of the cerebral cortex to the subthalamic nucleus, *The Journal of comparative neurology*, 236(1), pp. 14-28.

Baron, M.S., Sidibé, M., DeLong, M.R. & Smith, Y., 2001, Course of motor and associative pallidothalamic projections in monkeys, *The Journal of comparative neurology*, 429(3), pp. 490-501.

Beart, P.M., Summers, R.J., Stephenson, J.A., Cook, C.J. & Christie, M.J., 1990, Excitatory amino acid projections to the periaqueductal gray in the rat: a retrograde transport study utilizing D[3H]aspartate and [3H]GABA, *Neuroscience*, 34(1), pp. 163-76.

Beckstead, R.M., 1983, A reciprocal axonal connection between the subthalamic nucleus and the neostriatum in the cat, *Brain research*, 275(1), pp. 137-42.

Bell, K., Churchill, L. & Kalivas, P.W., 1995, GABAergic projection from the ventral pallidum and globus pallidus to the subthalamic nucleus, *Synapse (New York, N.Y.)*, 20(1), pp. 10-8.

Benabid, A.L., 2009, Targeting the caudal intralaminar nuclei for functional neurosurgery of movement disorders, *Brain research bulletin*, 78(2-3), pp. 109-12.

Bevan, M.D. & Bolam, J.P., 1995, Cholinergic, GABAergic, and glutamate-enriched inputs from the mesopontine tegmentum to the subthalamic nucleus in the rat, *The Journal of neuroscience : the official journal of the Society for Neuroscience*, 15(11), pp. 7105-20.

Bevan, M.D., Bolam, J.P. & Crossman, A.R., 1994, Convergent synaptic input from the neostriatum and the subthalamus onto identified nigrothalamic neurons in the rat, *The European journal of neuroscience*, 6(3), pp. 320-34.

Bevan, M.D., Hallworth, N.E. & Baufreton, J., 2007, GABAergic control of the subthalamic nucleus, *Progress in brain research*, 160, pp. 173-88.

Bostan, A.C., Dum, R.P. & Strick, P.L., 2010, The basal ganglia communicate with the cerebellum, *Proceedings of the National Academy of Sciences of the United States of America*, 107(18), pp. 8452-6.

Canteras, N.S., Shammah-Lagnado, S.J., Silva, B.A. & Ricardo, J.A., 1988, Somatosensory inputs to the subthalamic nucleus: a combined retrograde and anterograde horseradish peroxidase study in the rat, *Brain research*, 458(1), pp. 53-64.

Canteras, N.S., Shammah-Lagnado, S.J., Silva, B.A. & Ricardo, J.A., 1990, Afferent connections of the subthalamic nucleus: a combined retrograde and anterograde horseradish peroxidase study in the rat, *Brain research*, 513(1), pp. 43-59.

Carpenter, M.B. & Jayaraman, A., 1990, Subthalamic nucleus of the monkey: connections and immunocytochemical features of afferents, *Journal für Hirnforschung*, 31(5), pp. 653-68.

Carpenter, M.B. & Strominger, N.L., 1967, Efferent fibers of the subthalamic nucleus in the monkey. A comparison of the efferent projections of the subthalamic nucleus, substantia nigra and globus pallidus, *American Journal of Anatomy*, 121(1), pp. 41-71.

Carpenter, M.B., Baton, R.R., Carleton, S.C. & Keller, J.T., 1981a, Interconnections and organization of pallidal and subthalamic nucleus neurons in the monkey, *The Journal of comparative neurology*, 197(4), pp. 579-603.

Carpenter, M.B., Carleton, S.C., Keller, J.T. & Conte, P., 1981b, Connections of the subthalamic nucleus in the monkey, *Brain research*, 224(1), pp. 1-29.

Carpenter, M.B., Nakano, K. & Kim, R., 1976, Nigrothalamic projections in the monkey demonstrated by autoradiographic technics, *The Journal of comparative neurology*, 165(4), pp. 401-15.

Cebrián, C. & Prensa, L., 2010, Basal ganglia and thalamic input from neurons located within the ventral tier cell cluster region of the substantia nigra pars compacta in the rat, *The Journal of comparative neurology*, 518(8), pp. 1283-300.

Chiba, T. & Murata, Y., 1985, Afferent and efferent connections of the medial preoptic area in the rat: a WGA-HRP study, *Brain research bulletin*, 14(3), pp. 261-72.

Ciesielska, A., Mittermeyer, G., Hadaczek, P., Kells, A.P., Forsayeth, J. & Bankiewicz, K.S., 2011, Anterograde axonal transport of AAV2-GDNF in rat basal ganglia, *Molecular therapy : the journal of the American Society of Gene Therapy*, 19(5), pp. 922-7.

Degos, B., Deniau, J.M., Le Cam, J., Mailly, P. & Maurice, N., 2008, Evidence for a direct subthalamo-cortical loop circuit in the rat, *The European journal of neuroscience*, 27(10), pp. 2599-610.

Deniau, J.M., Hammond, C., Chevalier, G. & Feger, J., 1978, Evidence for branched subthalamic nucleus projections to substantia nigra, entopeduncular nucleus and globus pallidus, *Neuroscience letters*, 9(2-3), pp. 117-21.

Deschênes, M., Bourassa, J., Doan, V.D. & Parent, A., 1996, A single-cell study of the axonal projections arising from the posterior intralaminar thalamic nuclei in the rat, *The European journal of neuroscience*, 8(2), pp. 329-43.

DeVito, J.L., Anderson, M.E. & Walsh, K.E., 1980, A horseradish peroxidase study of afferent connections of the globus pallidus in Macaca mulatta, *Experimental brain research. Experimentelle Hirnforschung. Expérimentation cérébrale*, 38(1), pp. 65-73.

Dong, H., Petrovich, G.D. & Swanson, L.W., 2000, Organization of projections from the juxtacapsular nucleus of the BST: a PHAL study in the rat, *Brain research*, 859(1), pp. 1-14.

Féger, J., Bevan, M. & Crossman, A.R., 1994, The projections from the parafascicular thalamic nucleus to the subthalamic nucleus and the striatum arise from separate neuronal populations: a comparison with the corticostriatal and corticosubthalamic efferents in a retrograde fluorescent double-labelling study, *Neuroscience*, 60(1), pp. 125-32.

Gaytán, S.P. & Pásaro, R., 1998, Connections of the rostral ventral respiratory neuronal cell group: an anterograde and retrograde tracing study in the rat, *Brain research bulletin*, 47(6), pp. 625-42.

Gerfen, C.R., Staines, W.A., Arbuthnott, G.W. & Fibiger, H.C., 1982, Crossed connections of the substantia nigra in the rat, *The Journal of comparative neurology*, 207(3), pp. 283-303.

GLEES, P. & WALL, P.D., 1946, Fibre connections of the subthalamic region and the centro-median nucleus of the thalamus, *Brain : a journal of neurology*, 69(3), pp. 195-208.

Goto, M. & Swanson, L.W., 2004, Axonal projections from the parasubthalamic nucleus, *The Journal of comparative neurology*, 469(4), pp. 581-607.

Gradinaru, V., Mogri, M., Thompson, K.R., Henderson, J.M. & Deisseroth, K., 2009, Optical deconstruction of parkinsonian neural circuitry, *Science (New York, N.Y.)*, 324(5925), pp. 354-9.

Groenewegen, H.J. & Berendse, H.W., 1990, Connections of the subthalamic nucleus with ventral striatopallidal parts of the basal ganglia in the rat, *The Journal of comparative neurology*, 294(4), pp. 607-22.

Groenewegen, H.J., Berendse, H.W. & Haber, S.N., 1993, Organization of the output of the ventral striatopallidal system in the rat: ventral pallidal efferents, *Neuroscience*, 57(1), pp. 113-42.

Haber, S.N., Groenewegen, H.J., Grove, E.A. & Nauta, W.J., 1985, Efferent connections of the ventral pallidum: evidence of a dual striato pallidofugal pathway, *The Journal of comparative neurology*, 235(3), pp. 322-35.

Haber, S.N., Lynd-Balta, E. & Mitchell, S.J., 1993, The organization of the descending ventral pallidal projections in the monkey, *The Journal of comparative neurology*, 329(1), pp. 111-28.

Hammond, C., Rouzaire-Dubois, B., Féger, J., Jackson, A. & Crossman, A.R., 1983, Anatomical and electrophysiological studies on the reciprocal projections between the subthalamic nucleus and nucleus tegmenti pedunculopontinus in the rat, *Neuroscience*, 9(1), pp. 41-52.

Hattori, T., Fibiger, H.C. & McGeer, P.L., 1975, Demonstration of a pallido-nigral projection innervating dopaminergic neurons, *The Journal of comparative neurology*, 162(4), pp. 487-504.

Hazrati, L.N. & Parent, A., 1992a, Convergence of subthalamic and striatal efferents at pallidal level in primates: an anterograde double-labeling study with biocytin and PHA-L, *Brain research*, 569(2), pp. 336-40.

Hazrati, L.N. & Parent, A., 1992b, Differential patterns of arborization of striatal and subthalamic fibers in the two pallidal segments in primates, *Brain research*, 598(1-2), pp. 311-5.

Henkel, C.K., 1981, Afferent sources of a lateral midbrain tegmental zone associated with the pinnae in the cat as mapped by retrograde transport of horseradish peroxidase, *The Journal of comparative neurology*, 203(2), pp. 213-26.

Inase, M., Tokuno, H., Nambu, A., Akazawa, T. & Takada, M., 1999, Corticostriatal and corticosubthalamic input zones from the presupplementary motor area in the macaque monkey: comparison with the input zones from the supplementary motor area, *Brain research*, 833(2), pp. 191-201.

Jackson, A. & Crossman, A.R., 1981, Subthalamic projection to nucleus tegmenti pedunculopontinus in the rat, *Neuroscience letters*, 22(1), pp. 17-22.

Joel, D. & Weiner, I., 1997, The connections of the primate subthalamic nucleus: indirect pathways and the open-interconnected scheme of basal ganglia-thalamocortical circuitry, *Brain Res Brain Res Rev*, 23(1-2), pp. 62-78.

Jürgens, U., 1984, The efferent and afferent connections of the supplementary motor area, *Brain research*, 300(1), pp. 63-81.

Kanazawa, I., Marshall, G.R. & Kelly, J.S., 1976, Afferents to the rat substantia nigra studied with horseradish peroxidase, with special reference to fibres from the subthalamic nucleus, *Brain research*, 115(3), pp. 485-91.

Karachi, C., Yelnik, J., Tandé, D., Tremblay, L., Hirsch, E.C. & François, C., 2005, The pallidosubthalamic projection: an anatomical substrate for nonmotor functions of the subthalamic nucleus in primates, *Movement disorders : official journal of the Movement Disorder Society*, 20(2), pp. 172-80.

Kelly, R.M. & Strick, P.L., 2004, Macro-architecture of basal ganglia loops with the cerebral cortex: use of rabies virus to reveal multisynaptic circuits, *Progress in brain research*, 143, pp. 449-59.

Kim, R., Nakano, K., Jayaraman, A. & Carpenter, M.B., 1976, Projections of the globus pallidus and adjacent structures: an autoradiographic study in the monkey, *The Journal of comparative neurology*, 169(3), pp. 263-90.

Kita, H., 2007, Globus pallidus external segment, *Progress in brain research*, 160, pp. 111-33.

Kita, H. & Kitai, S.T., 1987, Efferent projections of the subthalamic nucleus in the rat: Light and electron microscopic analysis with the PHA-L method, *The Journal of comparative neurology*, 260(3), pp. 435-52.

Kita, H. & Kitai, S.T., 1994, The morphology of globus pallidus projection neurons in the rat: an intracellular staining study, *Brain research*, 636(2), pp. 308-19.

Lavoie, B. & Parent, A., 1994, Pedunculopontine nucleus in the squirrel monkey: projections to the basal ganglia as revealed by anterograde tract-tracing methods, *The Journal of comparative neurology*, 344(2), pp. 210-31.

Lee, H.J., Rye, D.B., Hallanger, A.E., Levey, A.I. & Wainer, B.H., 1988, Cholinergic vs. noncholinergic efferents from the mesopontine tegmentum to the extrapyramidal motor system nuclei, *The Journal of comparative neurology*, 275(4), pp. 469-92.

Leichnetz, G.R., Hardy, S.G. & Carruth, M.K., 1987, Frontal projections to the region of the oculomotor complex in the rat: a retrograde and anterograde HRP study, *The Journal of comparative neurology*, 263(3), pp. 387-99.

Marani, E., Heida, T., Lakke, E.A. & Usunoff, K.G., 2008, The subthalamic nucleus. Part I: development, cytology, topography and connections, *Advances in anatomy, embryology, and cell biology*, 198, pp. 1-113, vii.

Mascaro, M.B., Prosdócimi, F.C., Bittencourt, J.C. & Elias, C.F., 2009, Forebrain projections to brainstem nuclei involved in the control of mandibular movements in rats, *European journal of oral sciences*, 117(6), pp. 676-84.

Mathai, A. & Smith, Y., 2011, The corticostriatal and corticosubthalamic pathways: two entries, one target. So what? *Frontiers in systems neuroscience*, 5, p. 64.

McBride, R.L. & Larsen, K.D., 1980, Projections of the feline globus pallidus, *Brain research*, 189(1), pp. 3-14.

Miyachi, S., Lu, X., Imanishi, M., Sawada, K., Nambu, A. & Takada, M., 2006, Somatotopically arranged inputs from putamen and subthalamic nucleus to primary motor cortex, *Neuroscience research*, 56(3), pp. 300-8.

Miyashita, E. & Tamai, Y., 1989, Subcortical connections of frontal 'oculomotor' areas in the cat, *Brain research*, 502(1), pp. 75-87.

Miyata, M., 1986, Interconnections between the subthalamic nucleus and the cerebral cortex of the cat, *Neuroscience research*, 4(1), pp. 1-11.

Mizuno, N., Ueyama, T., Itoh, K., Satoda, T., Tashiro, T. & Shigemoto, R., 1988, Direct projections from the subthalamic nucleus of Luys to the spinal cord in the Japanese monkey, *Neuroscience letters*, 89(1), pp. 13-8.

Monakow, K.H., Akert, K. & Künzle, H., 1978, Projections of the precentral motor cortex and other cortical areas of the frontal lobe to the subthalamic nucleus in the monkey, *Experimental brain research. Experimentelle Hirnforschung. Expérimentation cérébrale*, 33(3-4), pp. 395-403.

Moriizumi, T. & Hattori, T., 1992, Separate neuronal populations of the rat globus pallidus projecting to the subthalamic nucleus, auditory cortex and pedunculopontine tegmental area, *Neuroscience*, 46(3), pp. 701-10.

Moriizumi, T., Nakamura, Y., Kitao, Y. & Kudo, M., 1987, Ultrastructural analyses of afferent terminals in the subthalamic nucleus of the cat with a combined degeneration and horseradish peroxidase tracing method, *The Journal of comparative neurology*, 265(2), pp. 159-74.

Nakamura, S. & Sutin, J., 1972, The pattern of termination of pallidal axons upon cells of the subthalamic nucleus, *Experimental neurology*, 35(2), pp. 254-64.

Nakano, K., Hasegawa, Y., Tokushige, A., Nakagawa, S., Kayahara, T. & Mizuno, N., 1990, Topographical projections from the thalamus, subthalamic nucleus and pedunculopontine tegmental nucleus to the striatum in the Japanese monkey, Macaca fuscata, *Brain research*, 537(1-2), pp. 54-68.

Nakano, K., Kayahara, T., Tsutsumi, T. & Ushiro, H., 2000, Neural circuits and functional organization of the striatum, *Journal of neurology*, 247, pp. 1-15.

Nambu, A., 2007, Globus pallidus internal segment, *Progress in brain research*, 160, pp. 135-50.

Nambu, A., 2011, Somatotopic organization of the primate Basal Ganglia, *Frontiers in neuroanatomy*, 5, p. 26.

Nambu, A., Takada, M., Inase, M. & Tokuno, H., 1996, Dual somatotopical representations in the primate subthalamic nucleus: evidence for ordered but reversed body-map transformations from the primary motor cortex and the supplementary motor area, *The Journal of neuroscience : the official journal of the Society for Neuroscience*, 16(8), pp. 2671-83.

Nambu, A., Tokuno, H. & Takada, M., 2002, Functional significance of the cortico-subthalamo-pallidal 'hyperdirect' pathway, *Neuroscience research*, 43(2), pp. 111-7.

Nauta, H.J. & Cole, M., 1978, Efferent projections of the subthalamic nucleus: an autoradiographic study in monkey and cat, *The Journal of comparative neurology*, 180(1), pp. 1-16.

Noda, T. & Oka, H., 1993, Projections of the anterior coronal gyrus to the subthalamic nucleus in the cat: a combined retrograde and anterograde WGA-HRP study, *Brain research*, 605(2), pp. 305-8.

Nomura, S., Mizuno, N. & Sugimoto, T., 1980, Direct projections from the pedunculopontine tegmental nucleus to the subthalamic nucleus in the cat, *Brain research*, 196(1), pp. 223-7.

Parent, A. & De Bellefeuille, L., 1983, The pallidointralaminar and pallidonigral projections in primate as studied by retrograde double-labeling method, *Brain research*, 278(1-2), pp. 11-27.

Parent, A. & Hazrati, L.N., 1993, Anatomical aspects of information processing in primate basal ganglia, *Trends in neurosciences*, 16(3), pp. 111-6.

Parent, A. & Hazrati, L.N., 1995, Functional anatomy of the basal ganglia. II. The place of subthalamic nucleus and external pallidum in basal ganglia circuitry, *Brain Res Brain Res Rev*, 20(1), pp. 128-54.

Parent, A. & Smith, Y., 1987, Organization of efferent projections of the subthalamic nucleus in the squirrel monkey as revealed by retrograde labeling methods, *Brain research*, 436(2), pp. 296-310.

Parent, A., Smith, Y., Filion, M. & Dumas, J., 1989, Distinct afferents to internal and external pallidal segments in the squirrel monkey, *Neuroscience letters*, 96(2), pp. 140-4.

Parent, M. & Parent, A., 2007, The microcircuitry of primate subthalamic nucleus, *Parkinsonism & related disorders*, 13 Suppl 3, pp. S292-5.

Prensa, L. & Parent, A., 2001, The nigrostriatal pathway in the rat: A single-axon study of the relationship between dorsal and ventral tier nigral neurons and the striosome/matrix striatal compartments, *The Journal of neuroscience : the official journal of the Society for Neuroscience*, 21(18), pp. 7247-60.

Ricardo, J.A., 1980, Efferent connections of the subthalamic region in the rat. I. The subthalamic nucleus of Luys, *Brain research*, 202(2), pp. 257-71.

Rico, A.J., Barroso-Chinea, P., Conte-Perales, L., Roda, E., Gómez-Bautista, V., Gendive, M., Obeso, J.A. & Lanciego, J.L., 2010, A direct projection from the subthalamic nucleus to the ventral thalamus in monkeys, *Neurobiology of disease*, 39(3), pp. 381-92.

Robertson, R.T. & Feiner, A.R., 1982, Diencephalic projections from the pontine reticular formation: autoradiographic studies in the cat, *Brain research*, 239(1), pp. 3-16.

Romanelli, P., Esposito, V., Schaal, D.W. & Heit, G., 2005, Somatotopy in the basal ganglia: experimental and clinical evidence for segregated sensorimotor channels, *Brain Res Brain Res Rev*, 48(1), pp. 112-28.

Romansky, K.V., Usunoff, K.G., Ivanov, D.P. & Hassler, R., 1980, Pallidosubthalamic projection in the cat. Electron microscopic study, *Anatomy and embryology*, 159(2), pp. 163-80.

Rouiller, E.M., Moret, V. & Liang, F., 1993, Comparison of the connectional properties of the two forelimb areas of the rat sensorimotor cortex: support for the presence of a premotor or supplementary motor cortical area, *Somatosensory & motor research*, 10(3), pp. 269-89.

Royce, G.J. & Laine, E.J., 1984, Efferent connections of the caudate nucleus, including cortical projections of the striatum and other basal ganglia: an autoradiographic and horseradish peroxidase investigation in the cat, *The Journal of comparative neurology*, 226(1), pp. 28-49.

Royce, G.J. & Mourey, R.J., 1985, Efferent connections of the centromedian and parafascicular thalamic nuclei: an autoradiographic investigation in the cat, *The Journal of comparative neurology*, 235(3), pp. 277-300.

Sadikot, A.F. & Rymar, V.V., 2009, The primate centromedian-parafascicular complex: anatomical organization with a note on neuromodulation, *Brain research bulletin*, 78(2-3), pp. 122-30.

Sadikot, A.F., Parent, A. & François, C., 1992, Efferent connections of the centromedian and parafascicular thalamic nuclei in the squirrel monkey: a PHA-L study of subcortical projections, *The Journal of comparative neurology*, 315(2), pp. 137-59.

Saper, C.B. & Loewy, A.D., 1982, Projections of the pedunculopontine tegmental nucleus in the rat: evidence for additional extrapyramidal circuitry, *Brain research*, 252(2), pp. 367-72.

Sato, F., Lavallée, P., Lévesque, M. & Parent, A., 2000a, Single-axon tracing study of neurons of the external segment of the globus pallidus in primate, *The Journal of comparative neurology*, 417(1), pp. 17-31.

Sato, F., Parent, M., Levesque, M. & Parent, A., 2000b, Axonal branching pattern of neurons of the subthalamic nucleus in primates, *The Journal of comparative neurology*, 424(1), pp. 142-52.

Scarnati, E., Gasbarri, A., Campana, E. & Pacitti, C., 1987, The organization of nucleus tegmenti pedunculopontinus neurons projecting to basal ganglia and thalamus: a retrograde fluorescent double labeling study in the rat, *Neuroscience letters*, 79(1-2), pp. 11-6.

Semba, K. & Fibiger, H.C., 1992, Afferent connections of the laterodorsal and the pedunculopontine tegmental nuclei in the rat: a retro- and antero-grade transport and immunohistochemical study, *The Journal of comparative neurology*, 323(3), pp. 387-410.

Shink, E., Bevan, M.D., Bolam, J.P. & Smith, Y., 1996, The subthalamic nucleus and the external pallidum: two tightly interconnected structures that control the output of the basal ganglia in the monkey, *Neuroscience*, 73(2), pp. 335-57.

Sloper, J.J., Brodal, P. & Powell, T.P., 1983, An anatomical study of the effects of unilateral removal of sensorimotor cortex in infant monkeys on the subcortical projections of the contralateral sensorimotor cortex, *Brain : a journal of neurology*, 106 (Pt 3), pp. 707-16.

Smith, Y., Bevan, M.D., Shink, E. & Bolam, J.P., 1998, Microcircuitry of the direct and indirect pathways of the basal ganglia, *Neuroscience*, 86(2), pp. 353-87.

Smith, Y., Bolam, J.P. & Von Krosigk, M., 1990a, Topographical and Synaptic Organization of the GABA-Containing Pallidosubthalamic Projection in the Rat, *The European journal of neuroscience*, 2(6), pp. 500-11.

Smith, Y., Hazrati, L.N. & Parent, A., 1990b, Efferent projections of the subthalamic nucleus in the squirrel monkey as studied by the PHA-L anterograde tracing method, *The Journal of comparative neurology*, 294(2), pp. 306-23.

Smith, Y., Wichmann, T. & DeLong, M.R., 1994, Synaptic innervation of neurones in the internal pallidal segment by the subthalamic nucleus and the external pallidum in monkeys, *The Journal of comparative neurology*, 343(2), pp. 297-318.

Spooren, W.P., Veening, J.G. & Cools, A.R., 1993, Descending efferent connections of the sub-pallidal areas in the cat: projections to the subthalamic nucleus, the hypothalamus, and the midbrain, *Synapse (New York, N.Y.)*, 15(2), pp. 104-23.

Stanton, G.B., Goldberg, M.E. & Bruce, C.J., 1988, Frontal eye field efferents in the macaque monkey: I. Subcortical pathways and topography of striatal and thalamic terminal fields, *The Journal of comparative neurology*, 271(4), pp. 473-92.

Steeves, J.D. & Jordan, L.M., 1984, Autoradiographic demonstration of the projections from the mesencephalic locomotor region, *Brain research*, 307(1-2), pp. 263-76.

Steininger, T.L., Rye, D.B. & Wainer, B.H., 1992, Afferent projections to the cholinergic pedunculopontine tegmental nucleus and adjacent midbrain extrapyramidal area in the albino rat. I. Retrograde tracing studies, *The Journal of comparative neurology*, 321(4), pp. 515-43.

Sugimoto, T. & Hattori, T., 1983, Confirmation of thalamosubthalamic projections by electron microscopic autoradiography, *Brain research*, 267(2), pp. 335-9.

Sugimoto, T. & Hattori, T., 1984, Organization and efferent projections of nucleus tegmenti pedunculopontinus pars compacta with special reference to its cholinergic aspects, *Neuroscience*, 11(4), pp. 931-46.

Sugimoto, T., Hattori, T., Mizuno, N., Itoh, K. & Sato, M., 1983, Direct projections from the centre median-parafascicular complex to the subthalamic nucleus in the cat and rat, *The Journal of comparative neurology*, 214(2), pp. 209-16.

Swanson, L.W., Mogenson, G.J., Simerly, R.B. & Wu, M., 1987, Anatomical and electrophysiological evidence for a projection from the medial preoptic area to the 'mesencephalic and subthalamic locomotor regions' in the rat, *Brain research*, 405(1), pp. 108-22.

Takada, M., Li, Z.K. & Hattori, T., 1987, Long descending direct projection from the basal ganglia to the spinal cord: a revival of the extrapyramidal concept, *Brain research*, 436(1), pp. 129-35.

Takada, M., Ng, G. & Hattori, T., 1986, Single pallidal neurons project both to the striatum and thalamus in the rat, *Neuroscience letters*, 69(3), pp. 217-20.

Takada, M., Nishihama, M.S., Nishihama, C.C. & Hattori, T., 1988, Two separate neuronal populations of the rat subthalamic nucleus project to the basal ganglia and pedunculopontine tegmental region, *Brain research*, 442(1), pp. 72-80.

Takada, M., Tokuno, H., Hamada, I., Inase, M., Ito, Y., Imanishi, M., Hasegawa, N., Akazawa, T., Hatanaka, N. & Nambu, A., 2001, Organization of inputs from cingulate motor areas to basal ganglia in macaque monkey, *The European journal of neuroscience*, 14(10), pp. 1633-50.

Tandé, D., Féger, J., Hirsch, E.C. & François, C., 2006, Parafascicular nucleus projection to the extrastriatal basal ganglia in monkeys, *Neuroreport*, 17(3), pp. 277-80.

Temel, Y., Blokland, A., Steinbusch, H.W. & Visser-Vandewalle, V., 2005, The functional role of the subthalamic nucleus in cognitive and limbic circuits, *Progress in neurobiology*, 76(6), pp. 393-413.

Tokuno, H., Takada, M., Ikai, Y. & Mizuno, N., 1994, Direct projections from the deep layers of the superior colliculus to the subthalamic nucleus in the rat, *Brain research*, 639(1), pp. 156-60.

Tripathi, A., Prensa, L., Cebrián, C. & Mengual, E., 2010, Axonal branching patterns of nucleus accumbens neurons in the rat, *The Journal of comparative neurology*, 518(22), pp. 4649-73.

Usunoff, K.G., Hassler, R., Romansky, K.V., Wagner, A. & Christ, J.F., 1982, Electron microscopy of the subthalamic nucleus in the baboon. II. Experimental demonstration of pallido-subthalamic synapses, *Journal für Hirnforschung*, 23(6), pp. 613-25.

Van Der Kooy, D. & Hattori, T., 1980, Single subthalamic nucleus neurons project to both the globus pallidus and substantia nigra in rat, *The Journal of comparative neurology*, 192(4), pp. 751-68.

van der Kooy, D., Hattori, T., Shannak, K. & Hornykiewicz, O., 1981, The pallido-subthalamic projection in rat: anatomical and biochemical studies, *Brain research*, 204(2), pp. 253-68.

Veening, J.G., Te Lie, S., Posthuma, P., Geeraedts, L.M. & Nieuwenhuys, R., 1987, A topographical analysis of the origin of some efferent projections from the lateral hypothalamic area in the rat, *Neuroscience*, 22(2), pp. 537-51.

Velayos, J.L. & Reinoso-Suárez, F., 1985, Prosencephalic afferents to the mediodorsal thalamic nucleus, *The Journal of comparative neurology*, 242(2), pp. 161-81.

Whittier, J.R. & Mettler, F.A., 1949, Studies on the subthalamus of the rhesus monkey. I. Anatomy and fiber connections of the subthalamic nucleus of Luys, *The Journal of comparative neurology*, 90(3), pp. 281-317.

Winn, P., 2006, How best to consider the structure and function of the pedunculopontine tegmental nucleus: evidence from animal studies, *Journal of the neurological sciences*, 248(1-2), pp. 234-50.

Wood, R.I. & Swann, J.M., 2005, The bed nucleus of the stria terminalis in the Syrian hamster: subnuclei and connections of the posterior division, *Neuroscience*, 135(1), pp. 155-79.

Woolf, N.J. & Butcher, L.L., 1986, Cholinergic systems in the rat brain: III. Projections from the pontomesencephalic tegmentum to the thalamus, tectum, basal ganglia, and basal forebrain, *Brain research bulletin*, 16(5), pp. 603-37.

Zahm, D.S., 1989, The ventral striatopallidal parts of the basal ganglia in the rat--II. Compartmentation of ventral pallidal efferents, *Neuroscience*, 30(1), pp. 33-50.

Zahm, D.S., Williams, E. & Wohltmann, C., 1996, Ventral striatopallidothalamic projection: IV. Relative involvements of neurochemically distinct subterritories in the ventral pallidum and adjacent parts of the rostroventral forebrain, *The Journal of comparative neurology*, 364(2), pp. 340-62.
